# Structure Elucidation of Purpurinidin from *Salix purpurea* Reveals an Undescribed Class of Pyranoanthocyanins—the Salicinocyanins

**DOI:** 10.1021/acs.jnatprod.5c00599

**Published:** 2025-09-26

**Authors:** Philipp Hopfstock, Mario Simirgiotis, Peter Winterhalter, Recep Gök

**Affiliations:** † 26527 Institute of Food Chemistry, Technische Universität Braunschweig, 38106 Braunschweig, Germany; ‡ Instituto de Farmacia, Facultad de Ciencias, Universidad Austral de Chile, Valdivia 5110566, Chile

## Abstract

Plants from the family Salicaceae have been used as health-promoting products for more than 3,500 years. They contain various secondary metabolites such as anthocyanins and phenolic glycosides (salicinoids) derived from salicin, a prodrug of salicylic acid, one of the most commonly used drugs today. Anthocyanins, which are primarily found in berries and certain vegetables, are recognized for their wide range of health benefits. Although salicinoids are well-known, knowledge regarding the occurrence of anthocyanins in this plant family is limited. In the early 1970s, Bridle et al. discovered an unknown anthocyanin in the bark of *Salix purpurea*, which was named purpurinidin. As far as we know, however, the structure of purpurinidin has not been elucidated to date. In this work, we present the isolation and structure elucidation of compound **1** that we suspect to be the aforementioned purpurinidin, which reveals the existence of new group of anthocyanin- and salicinoid-derived pyranoanthocyanin-type compounds. We have named this new type of pyranoanthocyanins “salicinocyanins” to emphasize their chemical origins.

## Graphical abstract

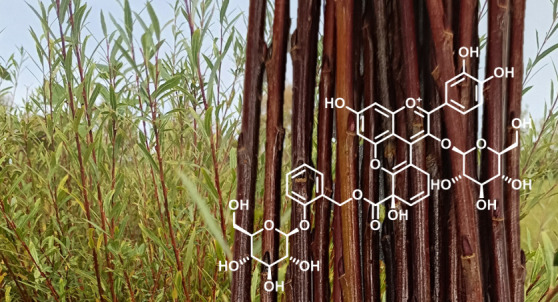

The Salicaceae family is mainly composed of *Salix* and *Populus* species and includes several hundred taxa and numerous naturally occurring hybrids.^1^ Plants of the genus *Salix* contain a wide range of biologically active compounds, such as salicin, a precursor of the well-known drug salicylic acid.^2^ Usage of *Salix* as a remedy for treatment of different health-related symptoms dates back more than 3,500 years.^3^ Generally, plants of this genus also contain various phenolic glucosides derived from salicin, which are classified as salicinoids.^4,5^ Salicinoids typically consist of salicin derivatives featuring structural modifications such as esterification with 1-hydroxy-6-oxo-2-cyclohexenecarboxylic acid (HCC), while the abbreviation HCH is used for the esterified form of HCC, which is often accompanied by additional substitutions, including acyl or organic acid groups, at specific positions of the glycosidic unit.^5^ This group of more complex salicinoids is expanding due to new discoveries in different plants of the Salicaceae family. The most recent finding was the discovery of new dimeric miyabeacin analogues by Noleto-Dias et al. (2024).^4,6–8^

A taxonomical characteristic of plants from the genus *Salix* is the variation in bark color, which ranges from green to yellow, red, and purple. Plant colors result from different pigments and secondary metabolites, primarily flavonoids, betalains, chlorophylls and carotenoids.^9^ Anthocyanins are a large group of water-soluble pigments commonly occurring in fruits and various vegetables.^10^ Although anthocyanins are biologically active and well-researched substances, data on anthocyanins in plants of the Salicaceae family are limited.^11,12^

Bridle et al. (1970) described the occurrence of cyanidin-, delphinidin-, and small amounts of petunidin-glucoside in different hybrids of *Salix purpurea*.^13^ Zhou et al. (2022) compared the metabolomic profiles of green, red, and purple barks from three different *Salix* species and identified seven different anthocyanins. Furthermore, a study by Alcalde-Eon et al. (2016) compared the anthocyanin contents in the catkins of three different species of the genus *Populus* L. and found several flavonol-anthocyanins.^9,14^ Another study described ten anthocyanins in the fruits of *Dovyalis hebecarpa*, a member of the Salicaceae family.^15^

The group of anthocyanins, characterized by an additional ring system (D-ring, pyran ring), is referred to as pyranoanthocyanins.^16^ Pyranoanthocyanins are found primarily in aged red wines and certain processed fruit and vegetable juices.^17^ Various pyranoanthocyanins have been identified to date, including vitisins, portisins, pinotins, and hydroxyphenyl-, methyl-, and vinylflavanol-pyranoanthocyanins, as well as pyranoanthocyanin dimers.^17–19^ Pyranoanthocyanins exhibit greater stability against bleaching and pH variations compared to their anthocyanin counterparts.^16,20^ Furthermore, the newly formed D-ring induces a hypsochromic shift relative to their anthocyanin precursors, resulting in a lower maximum absorption wavelength ranging from 478 to 510 nm.^18^ This shift imparts an orange hue, except in the case of portisins, which display a bathochromic shift toward 580 nm, resulting in a bluish color.^21^ The combination of increased stability and a broad color spectrum makes pyranoanthocyanins extremely attractive as dyes and pigments for industrial applications.^19^

A recent study by our group focused on the phenolic composition of the fruits of *Azara serrata* Ruiz&Pav. and revealed several cyanidin- and delphinidin-derivatives.^22^ Particularly noteworthy is that the berries contained a previously unidentified anthocyanin type that is inconsistent and is not explained by the extensive mass spectrometric literature data on anthocyanins. More specifically, these anthocyanins did not exhibit characteristic fragments by mass spectrometry, for example, [M]^+^ *m*/*z* 287 for cyanidin. The high-resolution mass-to-charge ratios and the fragmentation patterns suggested a condensation reaction between glycosylated anthocyanins and various salicinoids present in the berries. Consequently, we proposed that the double bond of HCH-group of the salicinoid can undergo a condensation reaction with the hydroxy group at position 5 of the A-ring and position 4 of the C-ring of the anthocyanin backbone, thereby forming a new heterocyclic pyran ring (cf. Figure 1).^22^ Our mechanistic proposal, in our opinion, is coherent with earlier analytical findings by Bridle et al. (1973), who first identified an unknown anthocyanin in the bark of *Salix purpurea* and suspected the presence of a dimeric anthocyanin species with a fructose and glucose moiety.^23^ This unknown anthocyanin was named purpurinidin, a designate that persists today, despite the fact that its structure remains unresolved.^2,23^

**Figure 1:**
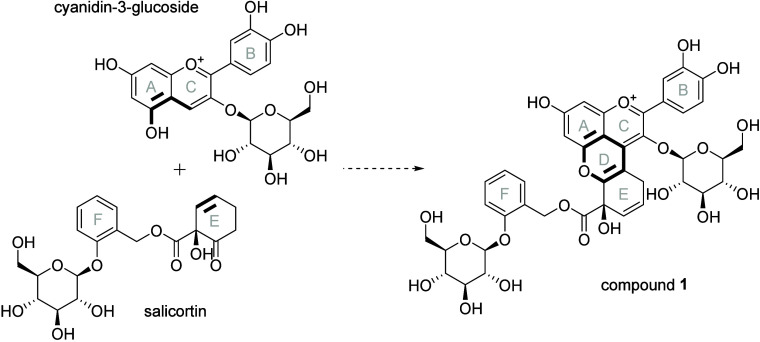
Proposed formation of a pyranoanthocyanin-type compound (compound **1**) in the presence of salicortin and cyanidin-3-glucoside. The linkage positions and pyran ring formed are marked in bold.

Here, we report the first isolation and structure elucidation of the natural compound presumably identified as purpurinidin from the bark of *Salix purpurea*. Based on the structure of compound **1** and the possible diversity of analogues, we have named this newly discovered group of anthocyanin- and salicinoid-derived compounds “salicinocyanins” to reflect the involvement of both precursor molecules. In addition, biomimetic formation experiments were conducted using a methanolic extract of *Salix purpurea* leaves and an enriched strawberry extract to prove the interaction between anthocyanins and salicinoids and to demonstrate the potential diversity of this newly discovered group of substances.

## Results and Discussion

The freeze-dried powdered bark of *Salix purpurea* (100 g) was processed following an established protocol for the enrichment of anthocyanins.^24^ Sartobind IEX MA 75 membrane extract from *Salix purpurea* bark was analyzed using an UHPLC-DAD-TIMS-TOF system to identify both known and unknown derivatives (cf. Figure 2; full table cf. Figure S1 and Table S1).

**Figure 2:**
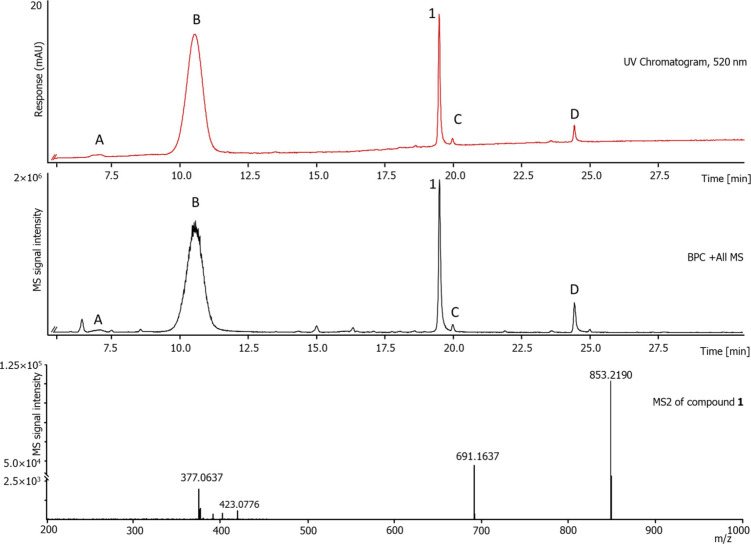
UHPLC-DAD-TIMS-TOF measurement of a *Salix purpurea* bark Sartobind IEX MA 75 membrane extract in ESI positive mode and the MS2 spectrum of compound **1**.

Among the five annotated anthocyanins, compound **B** was identified as cyanidin-3-glucoside based on comparison with a commercial standard (cf. Figure S2 and Table S2). Compound **A** was tentatively assigned as an (epi)-catechin-cyanidin glucoside derivate.^25^ Compounds **1**, **C**, and **D** were annotated as pyranoanthocyanins of an unknown structure. Based on their high-resolution mass spectra, they are likely structurally related to the compounds previously described in our recent study.^22^ High-resolution mass spectrometry of compound **1** revealed a molecular ion at [M]^+^ at *m*/*z* 853.2190, indicating the sum formula of C_41_H_41_O_20_^+^ with 22 double bond equivalents. The fragmentation pattern of compound **1** (cf. Figure 2) includes *m*/*z* 691 resulting from the hexose [M-hexose], *m*/*z* 423 from the further loss of the salicin moiety [M-salicin], and finally *m*/*z* 377 from the subsequent loss of formic acid [M-HCOOH]. Due to its relatively high abundance (14% of the peak area of cyanidin-3-glucoside), compound **1** was chosen for isolation. Isolation was performed using analytical HPLC, resulting in approximately 3.4 mg of compound **1** as a purple powder. UV/vis analysis of compound **1** (0.01% HCl–MeOH) showed an absorption maximum at 512 nm and a characteristic local UV maximum at 354 nm, consistent with a pyranoanthocyanin chromophore (cf. Figure S3).^18,26^ The recorded IR spectrum shows strong signals for C=C stretching vibrations (1589 cm^–1^) and O–H stretching (3389 cm^–1^), which is in agreement with the calculated sum formula and double bond equivalent of 22 (cf. Figure S4).

Based on the spectrometric and spectroscopic data as well as our recent findings regarding *Azara serrata*,^22^ we assume that compound **1** is derived from the two precursors cyanidin-3-glucoside and salicortin, which also was identified using a commercial standard and UHPLC-DAD-TIMS-TOF measurements (cf. Figure S5 and Table S3). Therefore, we conducted 1D and 2D NMR experiments (Figure 3, Table 1, and Table S4), confirming a pyranoanthocyanin-type structure of compound **1**. The NMR analyses were performed using the solvent mixture CH_3_OD/TFA-*d*_1_ (95/5 v/v with 0.01% TMS w/v) to maintain the substance in its cationic form during measurements.^27^ For the determination of the absolute configuration of the sugar units, compound **1** was hydrolyzed and subjected to chiral derivatization according to the method of Tanaka et al. (2007), with modifications described by Wang et al. (2012).^28,29^ The resulting sugar derivatives and the derivatized commercially available standards (d- and l-glucose) were analyzed by LC-MS.

**Figure 3:**
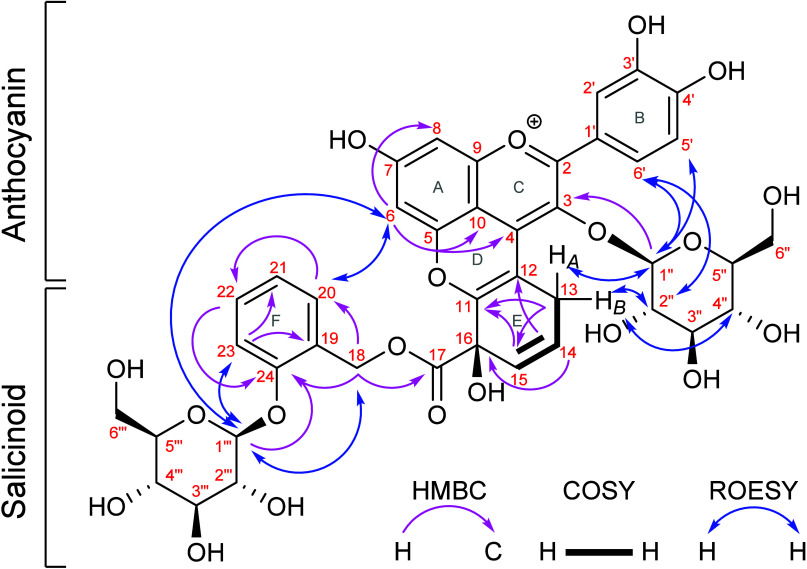
Structure of compound **1**, with key HMBC correlations (^1^H = 600 MHz, ^13^C = 151 MHz, CD_3_OD/TFA-*d*_1_ (95/5, v/v);^27^ containing TMS (0.01% w/v)).

**Table 1:** NMR Data (^1^H 600 MHz, ^13^C 151 MHz)^a^ of Compound **1**

position^b^	δ_C_, type	δ_H_ (*J* in Hz^c^)	HMBC
2	166.8 C		
3	136.8 C		
4	149.7 C		
5	153.5 C		
6	101.1 CH	6.86, d (2.0)	4, 5, 7, 8, 10
7	168.3 C		
8	101.1 CH	7.16, d (2.0)	6, 7, 9, 10
9	154.4 C		
10	110.2 C		
11	113.6 C		
12	163.4 C		
13	29.9 CH_2_	(*A*) 3.78, ddd (22.7, 3.6, 2.0)	11, 12, 14, 15
		(*B*) 4.24, ddd (22.7, 3.6, 2.3)	
14	125.0 CH	5.92, dt (10.0, 2.0)	12, 13, 16, 17
15	130.1 CH	6.34, dt (10.0, 3.6)	11, 13, 16, 17
16	73.6 C		
17	171.7 C		
18	64.9 CH_2_	(*A*) 5.35, d (12.2)	17, 19, 20, 24
		(*B*) 5.40, d (12.2)	
19	126.3 C		
20	131.1 CH	7.22, dd (7.5, 1.7)	18, 22, 23, 24
21	123.7 CH	6.93, td (7.5, 1.0)	19, 20, 22, 24
22	131.4 CH	7.24, ddd (8.3, 7.5, 1.7)	19, 20, 23, 24
23	117.4 CH	7.11, dd (8.3, 0.8)	18, 19, 21, 22, 24
24	157.4 C		
1′	121.6 C		
2′	118.2 CH	7.72, d (2.3)	2, 1′, 3′, 4′, 6′
3′	147.3 C		
4′	154.1 C		
5′	117.1 CH	6.98, d (8.6)	2, 1′, 3′, 4′
6′	126.8 CH	7.91, dd (8.6, 2.3)	2, 2′, 4′
1″	104.6 CH	4.56, d (7.8)	3, 3″, 5″
2″	75.1 CH	3.60, dd (9.3, 7.8)	1″, 3″
3″	77.4 CH	3.26, dd (9.0, 9.0)	1″, 2″, 4″
4″	71.5 CH	3.17, dd (9.8, 8.9)	3″, 4″, 5″, 6″
5″	79.1 CH	3.05, ddd (9.8, 6.6, 2.0)	1″, 3″, 4″, 6″
6″	62.5 CH_2_	(*A*) 3.38, dd (11.8, 6.6)	4″, 5″
		(*B*) 3.68, dd (11.8, 2.0)	
1′′′	103.3 CH	4.79, d (7.6)	24, 5′′′
2′′′	75.1 CH	3.37, dd (9.2, 7.6)	1′′′, 3′′′
3′′′	78.2 CH	3.42, dd (9.2, 8.7)	2′′′, 4′′′
4′′′	71.6 CH	3.29, dd (9.8, 8.7)	3′′′, 5′′′, 6′′′
5′′′	78.4 CH	3.36–3.39, m	1′′′, 4′′′, 6′′′
6′′′	62.6 CH_2_	(*A*) 3.63, dd (12.0, 5.9)	4′′′, 5′′′
		(*B*) 3.84, dd (12.0, 2.3)	

a Solvent: CD_3_OD/TFA-*d*_1_ (95/5, v/v); containing TMS (0.01% w/v) δ = 0.0 ppm for ^1^H and ^13^C.

b For numbering of the carbon atoms, refer to the chemical structure in Figure 3 (assignment of C–H via HSQC data).

c For CH_2_ groups with diastereotopic protons, (*A*) and (*B*) indicate the shielded and deshielded nucleus, respectively.

The protons of anthocyanin unit on rings A and C at positions H-6, H-8, H-2′, H-4′, H-5′, and H-6′ as well as protons of salicinoid at positions H-18, H-20, H-21, H-22, and H-23 can be assigned based on similarity to literature data.^7,8,30^ The two doublets at δ 4.56 and 4.79 ppm with coupling constants *J* 7.8 and 7.6 Hz, respectively, represent the two anomeric hydrogen atoms of two β-configured sugar units in the structure, while both anomeric hydrogen atoms show correlations in the HSQC-TOCSY to the H-2″–5″ and H-2′′′–5′′′, respectively, confirming their *trans-diaxial* relationship in the glucose units. The absolute configuration of the β-glucose units was determined to be the d-form by retention time comparison (17.23 min) confirmed by LC-MS (*m*/*z* 433) after derivatization, compared with l-glucose (16.24 min) and d-glucose (17.20 min), respectively (cf. Figure S6).^29^

Four CH_2_ groups, two of which belong to the glucose units, and a total of 25 hydrogen bearing as well as 16 non-hydrogen bearing carbon atoms can be derived from ^13^C and DEPT135 measurements. The ^13^C signal at δ 171.7 ppm was assigned to the ester function at position C-17 according to the HMBC from H-2–H-18­(*A*)/(*B*) at δ 5.35 and 5.40 ppm via ^3^ *J* coupling. A negative DEPT135 signal for C-14 (CH_2_), a downfield shifted ^13^C-signal for the keto group (C-15), and, most notably, the proton signals for H-11 and H-12 of the salicortin unit as well as the proton signal for H-4 of the anthocyanin unit are missing compared to literature data.^7,8,30^ The presence of a low intensity signal around δ 9.04 ppm indicates that a very small amount of free cyanidin-3-glucoside is present, due to acid-free processing during the final isolation step. However, the detection of a long-range ^4^ *J*_H6,C4_ coupling in the HMBC verifies the position of the non-hydrogen bearing nature of the C-4 carbon atom.

Thus, positions C-4, C-11, C-12, C-13, C-14, C-15, and C-16 represent key positions for the linkages to the pyran D- and condensed E-ring between the two units.

The coupling pattern and constants as well as the COSY correlations of protons in the salicinoid units H-13­(*A*)/(*B*), H-14, and H-15 indicate that the structure of the HCH group has changed in compound **1**. In the new spin system, protons H-14 and H-15 are *cis*-vinylic protons exhibiting a vicinal (^3^ *J*_*cis*_) coupling (*J* 10.0 Hz), whereas H-13­(*A*)/(*B*) are geminal protons showing a large geminal coupling (*J* 22.7 Hz). Carbon atom C-16 of the tertiary alcohol group of the HCH function was assigned to the signal at δ 73.6 ppm due to the anticipated upfield shift, the position of which has been confirmed by ^2,3^ *J* HMBC correlations from H-14 and H-15, respectively. The positions C-11 and C-12 were verified by ^3^ *J*-HMBC couplings from positions H-13, H-14, and H-15. The expected long-range correlation from salicinoid position H-13­(*A*)/(*B*) to anthocyanin position C-4 was not detected. This correlation was also not reported for vitisin B,^31^ which could be attributed to small ^1^H–^13^C ^3^ *J* coupling constants and a short *T*_*2*_ relaxation time of C-4 as reported by Voss et al. (2023).^32^

A ROESY experiment demonstrated the correlations of salicinoid protons to the glucose protons attached to the anthocyanin (cf. Table S4 and Figure 3) The ROESY cross signals enable differentiation of geminal protons H-13­(*A*)/(*B*), since H-13­(*A*) couples exclusively with the anomeric proton H-1″, while H-13­(*B*) couples with protons H-2″ and H-4″.

To the best of our knowledge, the novel salicinocyanins represent the first example in which an E-ring is incorporated into the fused ring system. In contrast to other pyranoanthocyanins such as those of the pinotin type, the E-ring is integrated through a C–C bond.^33,34^

Despite using three different established hydrolysis conditions (enzymatic, 2 M HCl in MeOH/H_2_O, and 2 M TFA) the proposed native aglycon with the sum formula of C_29_H_21_O_10_^+^ could not be detected by LC-MS.^35–37^ Instead, two artifacts with *m*/*z* 539.1183 (compound **C**) and *m*/*z* 377.0653 (compound **1a**), corresponding to the calculated sum formulas of C_27_H_23_O_12_^+^ and C_21_H_13_O_7_^+^, were observed (cf. Figure S7 and Table S5). The artifact **C** was already annotated in the membrane extract of *Salix purpurea* (cf. Figure 2) and appears to still contain one glucose unit, as indicated by its fragmentation to *m*/*z* 377. Following hydrolysis of compound **1**, 2.2 mg of the hydrolysis artifact, compound **1**a (*m*/*z* 377.0653, C_21_H_13_O_7_^+^), was obtained using the methodology applied for the isolation of compound **1** with slight modifications. High-resolution mass spectrometry provided a sum formula of C_21_H_13_O_7_^+^, corresponding to a double bond equivalent of 16, indicating the presence of an additional double bond in the system. The UV/vis spectrum of compound **1a** (0.01% HCl–MeOH) showed an absorption maximum at 546 nm and a local UV maximum at 381.5 nm (cf. Figure S8), consistent with the characteristics of pyranoanthocyanins. The IR spectrum displayed characteristic bands for C=C stretching vibrations (1599 cm^–1^) and O–H stretching (3361 cm^–1^), which is in agreement with the IR spectrum of compound **1** (cf. Figure S9).

NMR analyses of compound **1**a show that the E-ring remains integrated into the conjugated chromophore system (cf. Figure 4).

**Figure 4:**
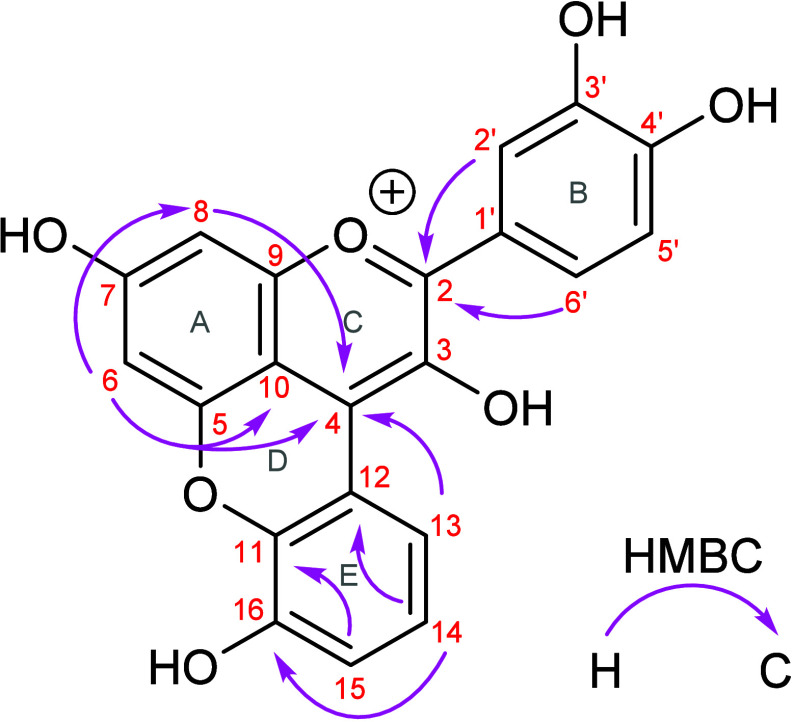
Structure of compound **1a**, with key HMBC correlations (^1^H = 600 MHz, ^13^C = 151 MHz, CD_3_OD/TFA-*d*_1_ (95/5, v/v);^27^ containing TMS (0.01% w/v)).

The data suggest that compound **1** underwent a decarboxylation at position C-16 as well as an aromatization of the E-ring during hydrolysis, which accounts for the increase in unsaturation. Table 2 shows the NMR assignments of compound **1**a.

**Table 2:** NMR Data (^1^H 600 MHz, ^13^C 151 MHz)^a^ of Compound **1**a

position^b^	δ_C_, type	δ_H_ (*J* in Hz)	HMBC
2	164.7 C		
3	139.5 C		
4	144.6 C		
5	153.2 C		
6	101.8 CH	7.14, d (2.0)	4, 5, 7, 8, 10
7	168.3 C		
8	99.3 CH	7.14, d (2.0)	4, 6, 7, 9, 10
9	155.0 C		
10	109.2 C		
11	145.8 C		
12	147.7 C		
13	123.0 CH	8.89, dd (8.4, 1.5)	4, 11, 12, 15, 16
14	126.3 CH	7.36, dd (8.3, 8.0)	11, 12, 13, 15, 16
15	124.0 CH	7.41, dd (7.9, 1.4)	11, 12, 13, 14, 16
16	118.0 C		
1′	121.6 C		
2′	118.2 CH	7.91, d (2.3)	2, 1′, 3′, 4′, 6′
3′	147.3 C		
4′	154.1 C		
5′	117.1 CH	7.04, d (8.6)	2, 1′, 2′, 3′, 4′
6′	126.8 CH	7.96, dd (8.6, 2.3)	2, 2′, 3′, 4′

a Solvent: CD_3_OD/TFA-*d*_1_ (95/5, v/v); containing TMS (0.01% w/v) δ = 0.0 ppm for ^1^H and ^13^C.

b For numbering of the carbon atoms, refer to the chemical structure in Figure 4 (assignment of C–H via HSQC data).

Biomimetic formation experiments were performed as a proof of concept to demonstrate the potential variety of the newly discovered pyranoanthocyanin species, utilizing an Amberlite XAD 7-HP strawberry extract in combination with a crude aqueous methanolic extract of *Salix purpurea* leaves following the method of Miyagusuku-Cruzado et al. (2021) as described in the Experimental Section.^38^ The enriched anthocyanin extract from strawberries consists primarily of six anthocyanins, four of which are expected to react with the salicinoids present in the leaf extract of *Salix purpurea* (cf. Figure S10 and Table S6). These anthocyanins include cyanidin-3-glucoside, and the tentatively identified pelargonidin-3-glucoside, pelargonidin-3-rutinoside, and pelargonidin-malonyl-glucoside.^39^ Given that the leaf extract contains salicortin, identified by a commercial standard, and the tentatively annotated tremulacin (benzoyl-salicortin) as potential reaction partners (cf. Figure S5 and Table S3), the formation of six novel pelargonidin-type salicinocyanins is anticipated (cf. Figure 5).

**Figure 5:**
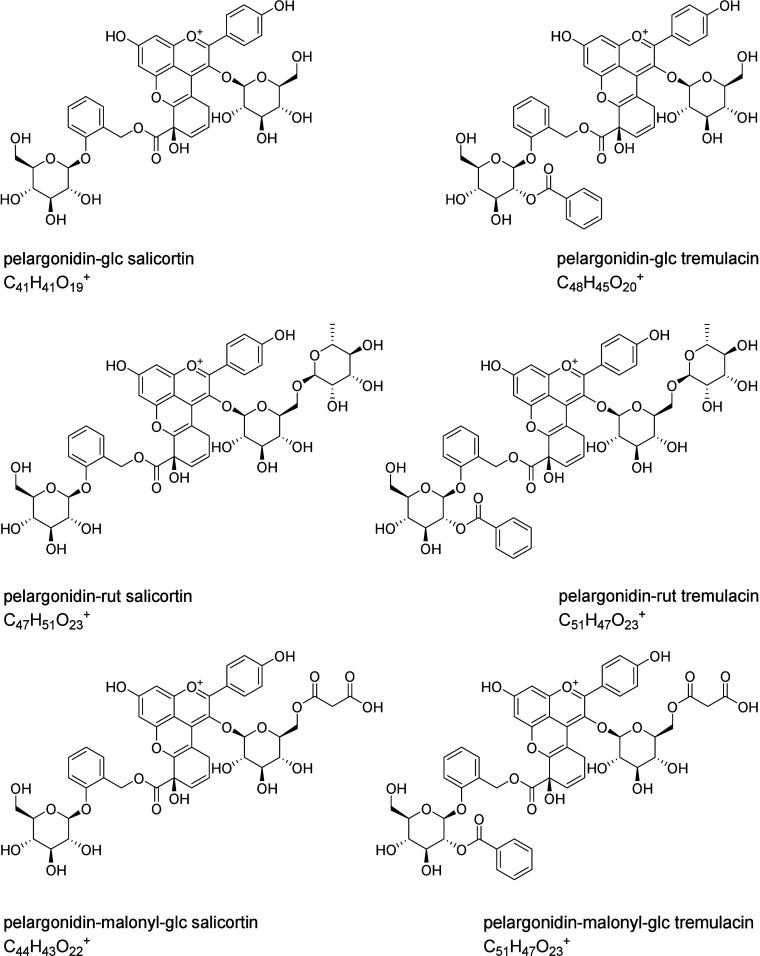
Proposed pelargonidin based salicyinocyanins formed during the biomimetic test (glc, glucose; rut, rutinose).

The main salicinocyanins detected were conjugates of pelargonidin-3-glucoside, which is the predominant anthocyanin in strawberries. The compound **1** analog of pelargonidin exhibited a mass-to-charge ratio of [M]^+^ *m*/*z* 837.2238 (C_41_H_41_O_19_^+^), with fragmentation patterns consistent with those observed for compound **1**. These fragments include the sequent loss of glucose, the salicin moiety, and formic acid, resulting in a core structure with an *m*/*z* of 361. Surprisingly, no significant hypsochromic shift was observed under the chosen chromatographic conditions for the newly formed compound (496 nm), as pelargonidin-3-glucoside has a UV maximum at 498 nm. It has been reported that pelargonidin-type pyranoanthocyanins exhibit a lower hypsochromic shift (8 nm) compared to pyranoanthocyanins derived from other anthocyanins.^40^ Also the chromatographic conditions, as well as low concentrations, may result in the observation of a low hypsochromic shift. However, similar to compound **1**, a new local absorption maximum at 358 nm was detected. Additionally, we annotated the salicortin conjugates of pelargonidin-rutinoside [M]^+^ *m*/*z* 983.2807 (C_47_H_51_O_23_^+^) and pelargonidin-malonyl-glucoside [M]^+^ *m*/*z* 923.2237 (C_44_H_43_O_22_^+^). The same pattern was observed for the tremulacin conjugates, with the pelargonidin-3-glucoside derivative being the most abundant. The fragmentation pattern of the other pyranoanthocyanins formed remains consistent with the loss of sugar moiety, salicinoid, and formic acid (cf. Table S6). Notably, no significant hypsochromic shift was detected for the tremulacin-type conjugates either.

The formation of pyranoanthocyanins during fermentation or storage is well documented and therefore a substantial portion of the literature describes their ex situ formation over time in red wines and other processed foods.^17,34,41^ These compounds have also been identified in juices derived from blood orange (*Cirtus sinensis* L.), black carrot (*Daucus carota* L.) and fermented bilberry (*Vaccinium myrtillus* L.) juice.^42–44^ Reports on the in situ formation of pyranoanthocyanins are comparatively scarce, with confirmed occurrences in red onion (*Allium cepa*), strawberry (*Fragaria ananassa*), staghorn sumac (*Rhus typhina* L.), North American ginseng (*Panax quinquefolius* L.), black currant (*Ribes nigrum*), *Azara serrata* Ruiz & Pav., and as presented herein, in *Salix purpurea*.^22,40,45–48^ The formation of salicinocyanins does not appear to be restricted to reactions occurring within plant tissue. Freshly peeled bark of a *Salix purpurea* plant, immersed in liquid nitrogen and immediately extracted and analyzed, showed the same chromatographic pattern and peak ratio (data not shown) as the sample obtained via the extraction method applied for enrichment; it is reasonable to assume that compound **1** is a natural product. It remains unclear whether the biosynthesis of salicinocyanins is actively regulated in situ as a response to external stimuli or the seasonal growth of *Salix purpurea*.

## Conclusion

In this study, we successfully isolated and structurally characterized compound **1** from the bark of *Salix purpurea*. On the basis of spectrometric data and historical reports, we propose with high confidence that this compound corresponds to purpurinidin, a previously uncharacterized anthocyanin first described by Bridle et al. in the early 1970s.^23^ Notably, purpurinidin represents the first isolated member of a previously unknown class of anthocyanin–salicinoid conjugates, which we named salicinocyanins. This compound class also represents the first report of pyranoanthocyanins incorporating an E-ring into the extended heterocyclic system. Preliminary in vitro formation experiments suggest a high degree of structural diversity within this compound class. Given the well documented biological activities of both salicinoids and anthocyanins, the newly discovered salicinocyanins represent an interesting target for future investigation into their potential dietary and pharmacological relevance.^12,49,50^

## Experimental Section

### General Experimental Procedures

Optical rotation for compound **1** (0.01% HCl–MeOH) was measured by using an Anton Paar MCP 150 (Anton Paar Group AG, Graz, Switzerland) polarimeter. UV/vis spectra of compound **1** (0.01% HCl–MeOH) and compound **1a** (0.01% HCl–MeOH) were recorded using a Jasco V-750 (JASCO Corporation, Tokyo, Japan). The operating software was Spectra Manager, Version 2.13.00 (JASCO Corporation, Tokyo, Japan).

IR spectra were recorded using a Bruker ALPHA FT-IR spectrometer operated using software OPUS, Version 7.0 (Bruker Optics GmbH & Co. KG, Ettlingen, Germany).

NMR experiments were performed on a Bruker Avance II 600 instrument (^1^H NMR: 600 MHz, ^13^C NMR: 151 MHz; Bruker, Rheinstetten, Germany). Samples were recorded at room temperature in a mixture of CD_3_OD/TFA-*d*_1_ (95/5, v/v); containing TMS (0.01% w/v) and referenced to δ = 0.0 ppm for ^1^H and ^13^C as an internal standard (residue solvent signal of CD_3_OD; δ = 49.1 ppm for ^13^C). All chemical shifts δ were reported in parts per million (ppm) and coupling constants *J* in hertz (Hz).

For high-resolution mass spectrometry, the system was a TIMS-TOF equipped with an electrospray ionization source (Bruker Daltonik, Bremen, Germany). The ESI-positive measurements settings were as follows: scan range, *m*/*z* 100–1350; inversed ion mobility range 1/k0, 0.55–1.90 V s cm^–2^; ramp time, 100 ms, capillary voltage, 4500 V; nebulizing gas pressure, 2.20 bar (N_2_); dry gas flow rate, 10 L min^–1^ (N_2_); nebulizer temperature, 220 °C; collision energy, 30 eV. To calibrate the mass spectrometer and trapped ion mobility the ESI-L Low Concentration Tuning Mix (Agilent Technologies, Waldbronn, Germany) was used. Instrument control was performed using Bruker Compass Hystar Version 6.2 and otofControl Version 6.2 (Bruker Daltonik, Bremen, Germany), while spectral data were processed and analyzed with Bruker Compass DataAnalysis Version 5.3 (Bruker Daltonik, Bremen, Germany).

For low-resolution mass spectrometry, a Bruker HCT Ultra Ion Trap equipped with an electrospray ionization source (Bruker Daltonik, Bremen, Germany) was used to determine the glucose configuration. The ESI-positive measurements settings were as follows: scan range, *m*/*z* 100–1500; capillary voltage, 3000 V; dry gas (N_2_) flow rate, 10 L min^–1^ (N_2_); nebulizer pressure, 50 psi (N_2_); nebulizer temperature, 365 °C. Instrument control was performed using Bruker Compass Hystar Version 3.2 (Bruker Daltonik, Bremen, Germany), while spectral data were processed and analyzed with Bruker Compass DataAnalysis Version 5.3 (Bruker Daltonik, Bremen, Germany).

The chromatographic analysis for high-resolution mass spectrometry was performed on an Agilent 1290 Infinity system (Agilent Technologies, Waldbronn, Germany), equipped with a binary solvent manager, an autosampler, a column heater, and a diode array detector. The separation was carried out on a C18 Kinetex core–shell column (2.1 mm inner diameter x 100 mm, 1.7 μm) (Aschaffenburg, Germany). The used mobile phases were (A) H_2_O containing 0.1% formic acid and (B) MeCN containing 0.1% formic acid with temperature set at 40 °C, a flow rate of 0.3 mL/min and an injection volume of 1 μL. The gradient was as follows: initial condition 3% B, at 15 min 12% B, at 30 min 35% B, at 35 min 97% B until 37 min, at 38 min 3% B until 40 min.

Chromatographic analysis for the determination of the glucose configuration were performed on an Agilent 1100 System (Agilent Technologies, Waldbronn, Germany), equipped with a binary pump, an autosampler, a column heater and diode array detector. The separation was carried out on a Luna C18 (2) column (2.1 mm i.d. × 150 mm, 3 μm) (Aschaffenburg, Germany). For mobile phases, (A) H_2_O containing 1% formic acid and (B) MeCN were used. Temperature was set at 25 °C, with a flow rate of 0.25 mL/min and an injection volume of 1 μL. The gradient was as follows: initial condition 20% B, at 30 min 30% B, at 35 min 95% B until 40 min, at 41 min 20% B until 51 min.

### Chemicals

For the isolation of compound **1** Amberlite XAD-7HP was purchased from Sigma-Aldrich Co. (St. Louis, MO, USA), and Sartobind S strong acidic cation exchanger MA 75 was obtained from Sartorius Stedim Biotech GmBH (Göttingen, Germany). Chemicals used for the isolation procedure were double deionized water (Nanopure, Werner GmbH, Leverkusen, Germany); methanol (HPLC grade), dichloromethane (HPLC grade), which were purchased from Fisher Scientific (Loughborough, U.K.); formic acid (ACS Reagent 99–100% purity), and ethyl acetate (HPLC grade) were purchased from VWR Chemicals (PA, USA); sodium chloride (≥99.5%. p.a., ACS, ISO), disodium hydrogen phosphate dihydrate (≥99.0%, p.a.), citric acid (≥99.5%, p.a.) were obtained from Carl Roth GmbH & Co. KG (Karlsruhe, Germany). For the isolation of compound **1**, water and formic acid were mentioned above and acetonitrile (HPLC grade) was purchased from Honeywell Specialty Chemicals (Seelze, Germany). The solvents used for the UHPLC-DAD-TIMS-TOF analyses were water (LC-MS grade) and acetonitrile (UHPLC-MS grade), purchased from TH. Geyer GmbH & Co. KG (Renningen, Germany) and formic acid (LC-MS grade) purchased from Fisher Scientific (Loughborough, UK). Methanol-*d*_4_ (99.96% D) containing 0.01% (w/v) tetramethylsilane (TMS) and trifluoroacetic acid-d_1_ (TFA, 99.5% D) used for NMR spectroscopic measurements were obtained from Deutero GmbH (Kastellaun, Germany). The reference substances for identification, cyanidin-3-glucoside chloride (≥98.00%) and salicortin (≥93.00%), were purchased from Phytolab (Vestenbergsgreuth, Germany). For the hydrolysis of compound **1**, hydrochloric acid (≥37%, p.a.) and trifluouroacetic acid (≥99%) were obtained from Sigma-Aldrich Co. (St. Louis, MO, USA) and Rapidase AR 2000 was ordered from DSM Food Specialties B.V. (Delft, Netherlands). l-Glucose (≥99%), d-glucose (≥96%), l-cysteine methyl ester hydrochloride (98%), phenyl isothiocyanate (≥99%), pyridine anhydrous (99.8%), as well as Discovery DSC-18 SPE Tubes (bed wt 500 mg, volume 6 mL) were ordered from Sigma-Aldrich Co. (St. Louis, MO, USA).

### Plant Material

Nine plants of *Salix purpurea* were ordered from a local tree nursery in October of 2024. The height of the plants was between 60 and 100 cm. The taxonomic identity of *Salix purpurea* was confirmed by PD Dr. Gregor Aas (Ecological-Botanical Garden, University of Bayreuth, Germany). The *Salix purpurea* scrubs were cropped manually using secateurs, leaves were removed, and the bark was peeled off using a kitchen knife. The bark was then immediately freeze-dried and afterward pulverized using a laboratory mill (IKA-Werke, Staufen im Breisgau, Germany). The powder was stored in a dark place until use for the isolation as well as preparation of a methanolic extract. For biomimetic formation of the salicinocyanins, frozen strawberries were bought from a local market, freeze-dried, and pulverized using a laboratory mill. The same procedure was performed for leaves of *Salix purpurea* bushes. Both the strawberry powder and leaf powder were stored in a dark place at room temperature.

### Isolation of Compound **1**

For the isolation of compound **1**, 100 g of the freeze-dried pulverized *Salix purpurea* bark was macerated two times for 48 h in a dark place at room temperature by using a mixture of MeOH and H_2_O (80/20; v/v) containing 1% formic acid. The combined extracts were filtered, and the MeOH was removed using a rotary evaporator at 200 mbar and 40 °C. The further extraction procedure was performed according to Hopfstock et al. (2024), with modifications considering the solvents volumes accounting for the higher amount of plant material.^24^ CH_2_Cl_2_ (3 × 100 mL) was used, followed by EtOAc (3 × 200 mL). For adsorption on Amberlite XAD-7HP, a preconditioned column (i.d. = 5 cm) with a bed volume of 320 mL was used, and the resin was washed with H_2_O (3 × 320 mL) after sample loading. Afterward, the retained compounds were eluted using MeOH (2 × 320 mL). After removal of the MeOH using a rotary evaporator at 200 mbar and 40 °C, the extract was freeze-dried yielding 5.44 g of a red powder. For the Sartobind IEX MA 75 membrane roughly 1.5 g of the extract was used in portions of 250 mg. After removal of sodium chloride from the anthocyanin fraction, the sample was freeze-dried, yielding 62.5 mg of purified anthocyanin extract as powder. For isolation of compound **1** 62.5 mg purified anthocyanin extract was dissolved in 400 μL MeOH/H_2_O (1/1; v/v) containing 1% formic acid. The isolation was performed using a Phenomenex Luna C18 (4.60 mm i.d. × 250 mm, 5 μm) analytical column and an Agilent 1260 HPLC system equipped with an autosampler, column heater, and diode array detector. Chromatographic parameters were (A) H_2_O and (B) MeCN as mobile phases with the gradient being as follows: initial condition at 14% B until 3 min, at 5 min 18% B, at 11 min 22% B, at 14 min 25% B until 20 min, at 23 min 95% B until 25 min, at 26 min 14% B until 30 min. Flow rate was set to 1.0 mL min^–1^, injection volume was set to 25 μL and column temperature was set to 25 °C. The organic solvent of the fraction containing the purified compound **1** was removed using a rotary evaporator at 200 mbar and 40 °C. Afterward the fraction was diluted with H_2_O and freeze-dried, yielding 3.4 mg of the purified compound **1** which was stored at 4 °C until measured by NMR.

### Acid Hydrolysis of Compound **1**

Approximately 1 mg of compound **1** was dissolved in 500 μL of 2 M TFA and heated to 90 °C for 2 h. The hydrolysate was diluted with 2 mL of MeOH and dried under N_2_, then redissolved in 750 μL of H_2_O and loaded onto a preconditioned C18 SPE cartridge, followed by washing with 2 mL of H_2_O. The aqueous fraction was retained for the determination of the sugar moieties. The sample was washed with additional 10 mL of H_2_O and the hydrolyzed compounds were eluted using 10 mL of MeOH containing 100 μL of 2 M TFA.

### Derivatization of the Sugars of Compound **1**

The aqueous fraction from the SPE cleanup of the hydrolysate was diluted with 5 mL of MeOH and dried under N_2_. Subsequently, following the method described by Wang et al. (2017), the residue was dissolved in a solution of 120 μL of 0.3 mmol·mL^–1^ l-cysteine methyl ester in pyridine and treated for 1 h at 90 °C. After cooling, 160 μL of 0.69 mmol·mL^–1^ phenyl isothiocyanate in pyridine was added and the mixture was incubated for 1 h at 90 °C.^29^ After cooling, the sample was diluted 1:10 (v/v) with MeOH and analyzed by LC-MS. Approximately 1 mg of d- and l-glucose were treated the same manner.

### Isolation of Compound **1a**

For isolation of the hydrolysis artifact, compound **1a**, approximately 70 mg of membrane extract of freeze-dried pulverized *Salix purpurea* bark was hydrolyzed in 2 mL of 2 M TFA at 90 °C for 2 h. The hydrolysate was filtered, diluted with 5 mL of MeOH and evaporated at room temperature under a N_2_ stream. The residue was redissolved in 500 μL of H_2_O/MeOH (50/50; v/v) containing 1% formic acid. Further isolation was performed as described for the isolation of compound **1** using a slightly modified gradient, as follows: mobile phases (A) H_2_O and (B) MeCN initial condition 20% B until 5 min, at 16 min 25% B, at 19 min 32% B, at 20 min 95% B until 24 min, at 25 min 20% B until 30 min. After removal of MeCN and freeze-drying, approximately 2.2 mg of compound **1**a was obtained, which was stored at 4 °C until NMR measurement.

#### Compound **1**

Dark purple powder; [α]^25^ _d_ (*c* 0.02, MeOH + 0.01% HCl) + 180; UV (MeOH + 0.01% HCl) λmax (log ε) 286 (4.05), 354 (sh, 3.74) 514 (4.28) nm; FTIR (neat) ν_max_ 3389, 1590, 1352, 1068 cm^–1^; ESI-TOFMS *m*/*z* 853.2190 [M]^+^ (calcd for C_41_H_41_O_20_^+^, 853.2186); for ^1^H and ^13^C NMR data, see Table 1.

#### Compound **1a**

Dark purple powder; UV (MeOH + 0.01% HCl) λmax (log ε) 285 (4.00), 382 (sh, 3.60) 546 (4.22) nm; FTIR (neat) ν_max_ 3361, 1599, 1521, 1447, 1333 cm^–1^; ESI-TOFMS *m*/*z* 377.0653 [M]^+^ (calcd for C_21_H_13_O_7_^+^, 377.0656); for ^1^H and ^13^C NMR data, see Table 2.

### Biomimetic Formation of Salicinocyanins

The experiment for salicinocyanin formation was performed following the method described by Miyagusuku-Cruzado et al. (2021) with slight modifications.^38^ 100 mg of a freeze-dried *Salix purpurea* leaf extract (MeOH/H_2_O; 80/20; v/v + 1% formic acid) was combined with 10 mg of powdered freeze-dried strawberry Amberlite XAD-7HP resin extract and dissolved in 1 mL of 1 mM hydrochloric acid (pH 3) containing 0.1% (w/v) sodium benzoate and potassium sorbate.^38^ The mixture was kept in the dark at 40 °C for 7 days. Afterward the mixture was treated with the same analytical membrane chromatography protocol as described above.

## Supplementary Material



## Data Availability

The NMR data for compounds **1** and **1a** have been deposited in the Natural Products Magnetic Resonance Database (NP-MRD; www.np-mrd.org) and can be found at NP0351323 (compound **1**) and NP0351474 (compound **1a**).

## Notes

Spectroscopic and spectrometric data (PDF)
