# Supplementary material for: Structure Elucidation of Purpurinidin from Salix purpurea Reveals an Undescribed Class of Pyranoanthocyanins—the Salicinocyanins
Source: J Nat Prod. 2025 Sep 26;88(10):2333–41. doi: 10.1021/acs.jnatprod.5c00599 (PMC12560080; doi:10.1021/acs.jnatprod.5c00599)
Supplement: Supplementary file 1 [file np5c00599_si_001.pdf]

Supporting Information

Structure Elucidation of Purpurinidin from *Salix purpurea* Reveals an Undescribed Class of Pyranoanthocyanins – the Salicinocyanins

Philipp Hopfstock[1], Mario Simirgiotis[2], Peter Winterhalter[1], Recep Gök[1*]

[1] Institute of Food Chemistry, Technische Universität Braunschweig, 38106 Braunschweig, Germany

[2] Instituto de Farmacia, Facultad de Ciencias, Universidad Austral de Chile, 5110566 Valdivia, Chile

[*] Corresponding author

Dr. Recep Gök

Technische Universität Braunschweig
Institute of Food Chemistry
Schleinitzstrasse 20,
38106 Brauschweig, Germany
phone: +49 531 391 7205
fax: +49 531 391 7230
e-mail: r.goek@tu-braunschweig.de
https://orcid.org/0000-0002-1231-8679

Contents

Figures

Figure S1. UHPLC-DAD-TIMS-TOF measurement of a *Salix purpurea* bark Sartobind® IEX MA 75 membrane extract in ESI positive mode. ……………………………………………………………………………....……4

Figure S2. UHPLC-DAD-TIMS-TOF measurement of a cyanidin-3-glucoside (top) and a MeOH/H_2O (50/50, *v/v*) + 1 % formic acid extract of *Salix purpurea* bark (bottom) in ESI positive mode. …………..……………………5

Figure S3. UV/Vis spectrum of compound **1** in 0.01 % HCl-MeOH. ……………………………………………6

Figure S4. FT-IR spectrum of compound **1**. …………………………………………………………………………7

Figure S5. UHPLC-DAD-TIMS-TOF measurement of salicortin (top), MeOH/H_2O (50/50, *v/v*) + 1 % formic acid extract of *Salix purpurea* bark (middle) and MeOH/H_2O (50/50, *v/v*) + 1 % formic acid extract of *Salix purpurea* leaves (bottom) in ESI negative mode. ……………………………………………………………………………..8

Figure S6. Extracted ion chromatograms (*m/z* 433 +/- 0.5) of derivatized hydrolysate of compound **1** (top), derivatized hydrolysate + derivatized D-glucose (1:1) (middle) and derivatized hydrolysate of compound **1** + derivatized L-glucose (1:1) (bottom) measured in ESI positive mode. ……………………………………………11

Figure S7. UHPLC-DAD-TIMS-TOF measurement of a the hydrolysate of compound **1** by 2M TFA for 2 h and 90 °C in ESI positive mode and MS2 spectrum of compound **1**a. …………………………………………………12

Figure S8. UV/Vis spectrum of compound **1**a in 0.01 % HCl-MeOH. …………………………………………13

Figure S9. FT-IR spectrum of compound **1**a. ………………………………………………………………………14

Figure S10. UHPLC-DAD-TIMS-TOF measurement of a strawberry Amberlite™ XAD-7HP extract (left) and combined extract of *Salix purpurea* leaf and strawberry extract after biomimetic formation experiments and purification by Sartobind® IEX MA 75 membrane (right) in ESI positive mode. …………………………………15

Figure S11. ^{1}H NMR spectrum (CD_3OD/TFA-d, 19:1 *v/v*, 600 MHz) of compound **1**. The top panel shows the full spectrum, while the bottom panel displays a zoomed-in section highlighted by a red box. ………………………..16

Figure S12. ^{13}C NMR spectrum (CD_3OD/TFA-d, 19:1 *v/v*, 151 MHz) of compound **1**. The top panel shows the full spectrum, while the bottom panel displays a zoomed-in section highlighted by a red box. …………………...17

Figure S13. DEPT135 spectrum (CD_3OD/TFA-d, 19:1 *v/v*) of compound **1**. ……………………………………18

Figure S14. COSY spectrum (CD_3OD/TFA-d, 19:1 *v/v*) of compound **1**. ……………………………………...19

Figure S15. HSQC spectrum (CD_3OD/TFA-d, 19:1 *v/v*) of compound **1**. ………………………………………20

Figure S16. HMBC spectrum (CD_3OD/TFA-d, 19:1 *v/v*) of compound **1**. ……………………………………….21

Figure S17. HSQC-TOCSY spectrum (CD_3OD/TFA-d, 19:1 *v/v*) of compound **1**. ……………………………22

Figure S18. ROESY spectrum (CD_3OD/TFA-d, 19:1 *v/v*) of compound **1**. ……………………………………23

Figure S19. ^{1}H NMR spectrum (CD_3OD/TFA-d, 19:1 *v/v*, 600 MHz) of compound **1**a. The top panel shows the full spectrum, while the bottom panel displays a zoomed-in section highlighted by a red box. …………………...24

Figure S20. ^{13}C NMR spectrum (CD_3OD/TFA-d, 19:1 v/v, 151 MHz) of compound **1***a*. The top panel shows the full spectrum, while the bottom panel displays a zoomed-in section highlighted by a red box. …………………...25

Figure S21. DEPT135 spectrum (CD_3OD/TFA-d, 19:1 *v/v*) of compound **1**a. …………………………………...26

Figure S22. COSY spectrum (CD_3OD/TFA-d, 19:1 *v/v*) of compound **1**a. ……………………………………27

Figure S23. HSQC spectrum (CD_3OD/TFA-d, 19:1 *v/v*) of compound **1**a. ……………………………………28

Figure S24. HMBC spectrum (CD_3OD/TFA-d, 19:1 *v/v*) of compound **1**a. …………………………………...29

Tables

Table S1. Results of the UHPLC-DAD-TIMS-TOF measurement of a Salix purpurea bark Sartobind® IEX MA 75 membrane extract in ESI positive mode (cf. Figure S1). ……………………………………………………...4

Table S2. Results of the UHPLC-DAD-TIMS-TOF measurement of a cyanidin-3-glucoside (top) and a MeOH/H_2O (50/50, *v/v*) + 1 % formic acid extract of *Salix purpurea* bark (bottom) in ESI positive mode (cf. Figure S2). ………………………………………………………………………………………………5

Table S3. Results of the UHPLC-DAD-TIMS-TOF measurement of salicortin, MeOH/H_2O (50/50, *v/v*) + 1 % formic acid extract of *Salix purpurea* bark and MeOH/H_2O (50/50, *v/v*) + 1 % formic acid extract of *Salix purpurea* leafs in ESI negative mode (cf. Figure S5). ………………………………………………………………...8

Table S4. Full table of NMR data (^{1}H 600 MHz, ^{13}C 151 MHz)[a] of compound **1**. ………………………………9

Table S5. Results of the UHPLC-DAD-TIMS-TOF measurement of a the hydrolysate of compound **1** by 2M TFA for 2 h and 90 °C in ESI positive mode (cf. Figure S7). ………………………………………………………...12

Table S6. Results of the UHPLC-DAD-TIMS-TOF measurement of a strawberry Amberlite™ XAD-7HP extract and combined extract of Salix purpurea leaf and strawberry extract after biomimetic formation experiments and purification by Sartobind® IEX MA 75 membrane in ESI positive mode (cf. Figure S10). ………………………15

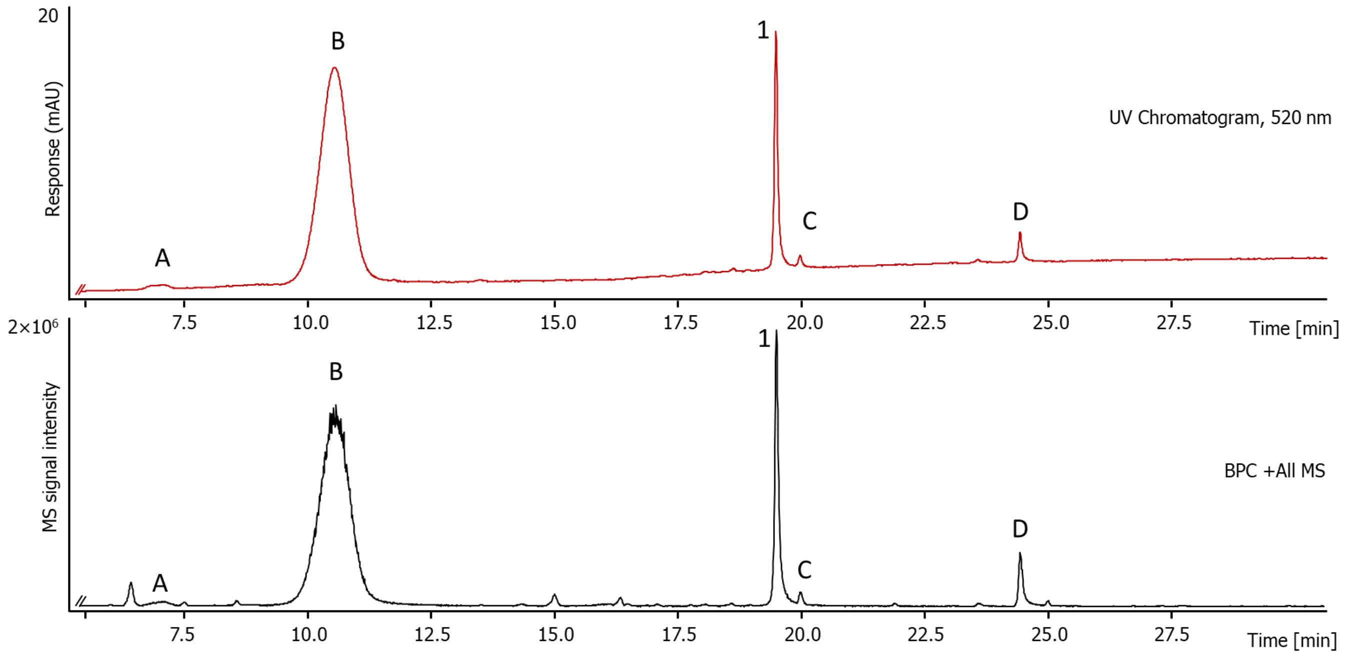

Figure S1. UHPLC-DAD-TIMS-TOF measurement of a *Salix purpurea* bark Sartobind® IEX MA 75 membrane extract in ESI positive mode.

Table S1. Results of the UHPLC-DAD-TIMS-TOF measurement of a Salix purpurea bark Sartobind® IEX MA 75 membrane extract in ESI positive mode (cf. Figure S1).

**compound Number**	**Retention Time [min]**	**Compound Name**	**UV/Vis max. [nm]**	**Molecular Formula**	**Theoretical Mass [*m/z*]**	**Detected Mass [*m/z*]**	**Mass Error[ppm]**	**Fragment Ion MS/MS [*m/z*]**	**CCS [Å^2]**
**A**	6.74	cyanidin-glucoside-(epi)catechin-derivate	n.d.	$C_{36}H_{33}O_{17}^+$	737.1712	737.1711	0.2	575/423/329/287	266.8
**B**	10.52	cyanidin-glucoside	278-514	$C_{21}H_{21}O_{11}^+$	449.1078	449.1073	1.1	287	202.6
**1**	19.52	compound **1**	352-504	$C_{41}H_{41}O_{20}^+$	853.2186	853.2190	-0.5	691/423/377	268.2
**C**	19.99	cyanidin-salicortin aglycon species	n.d.	$C_{27}H_{23}O_{12}^+$	539.1184	539.1182	0.4	377	220.2
**D**	24.44	cyanidin-glucoside-tremulacin	348-504	$C_{48}H_{45}O_{21}^+$	957.2448	957.2450	-0.2	795/423/377	287.5

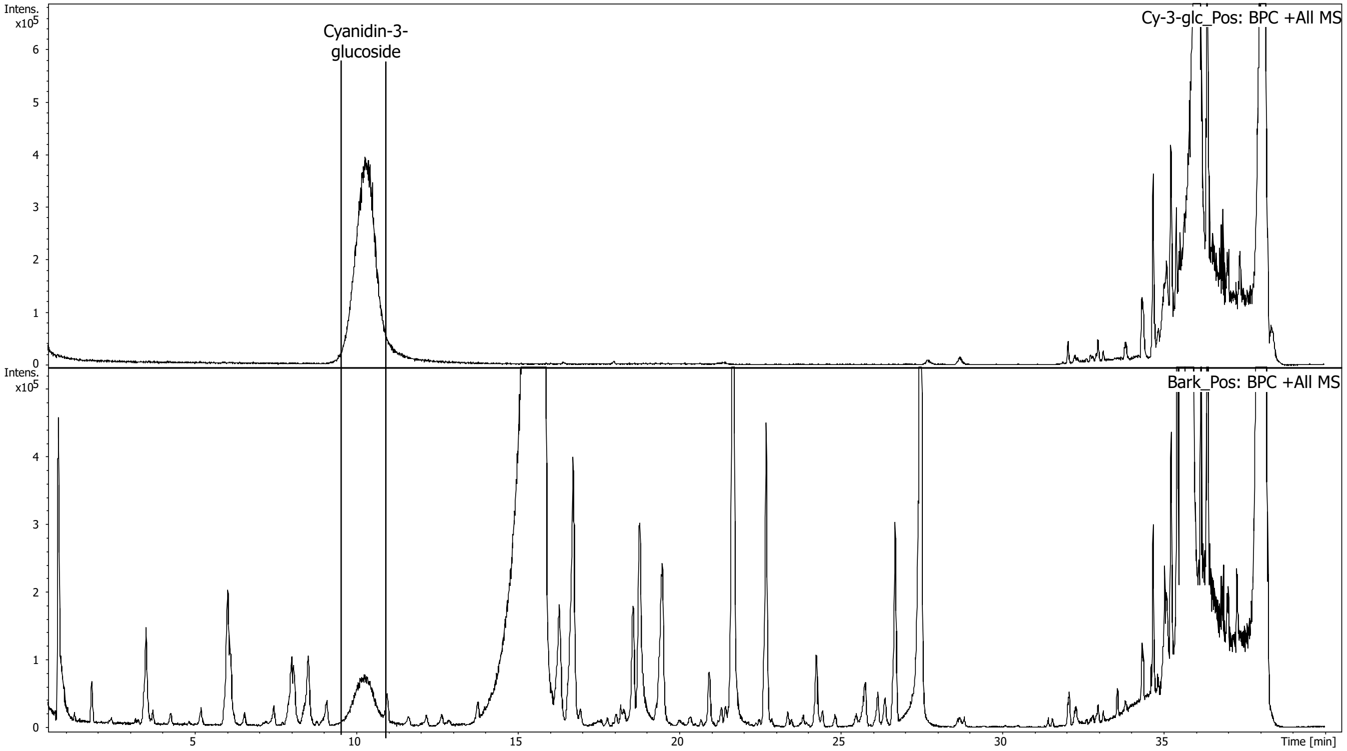

Figure S2. UHPLC-DAD-TIMS-TOF measurement of a cyanidin-3-glucoside (top) and a MeOH/H_2O (50/50, *v/v*) + 1 % formic acid extract of *Salix purpurea* bark (bottom) in ESI positive mode.

Table S2. Results of the UHPLC-DAD-TIMS-TOF measurement of a cyanidin-3-glucoside (top) and a MeOH/H_2O (50/50, *v/v*) + 1 % formic acid extract of *Salix purpurea* bark (bottom) in ESI positive mode (cf. Figure S2).

Retention Time [min]	Matrix	Molecular Formula	Theoretical Mass [*m/z*]	Detected Mass [*m/z*]	Mass Error [ppm]	Fragment Ion MS/MS [*m/z*]	CCS [Å2]
10.23	reference	$C_{21}H_{21}O_{11}$+	449.1078	449.1078	0.1	287	202.7
10.20	*S. purpurea* bark	$C_{21}H_{21}O_{11}$+	449.1078	449.1076	0.4	287	202.5

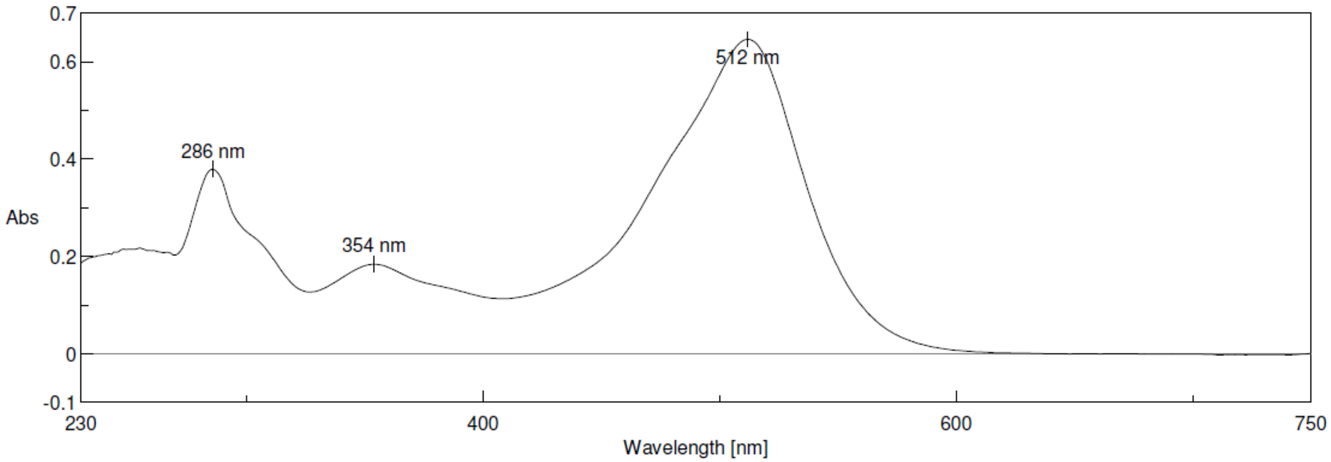

Figure S3. UV/Vis spectrum of compound **1** in 0.01 % HCl-MeOH.

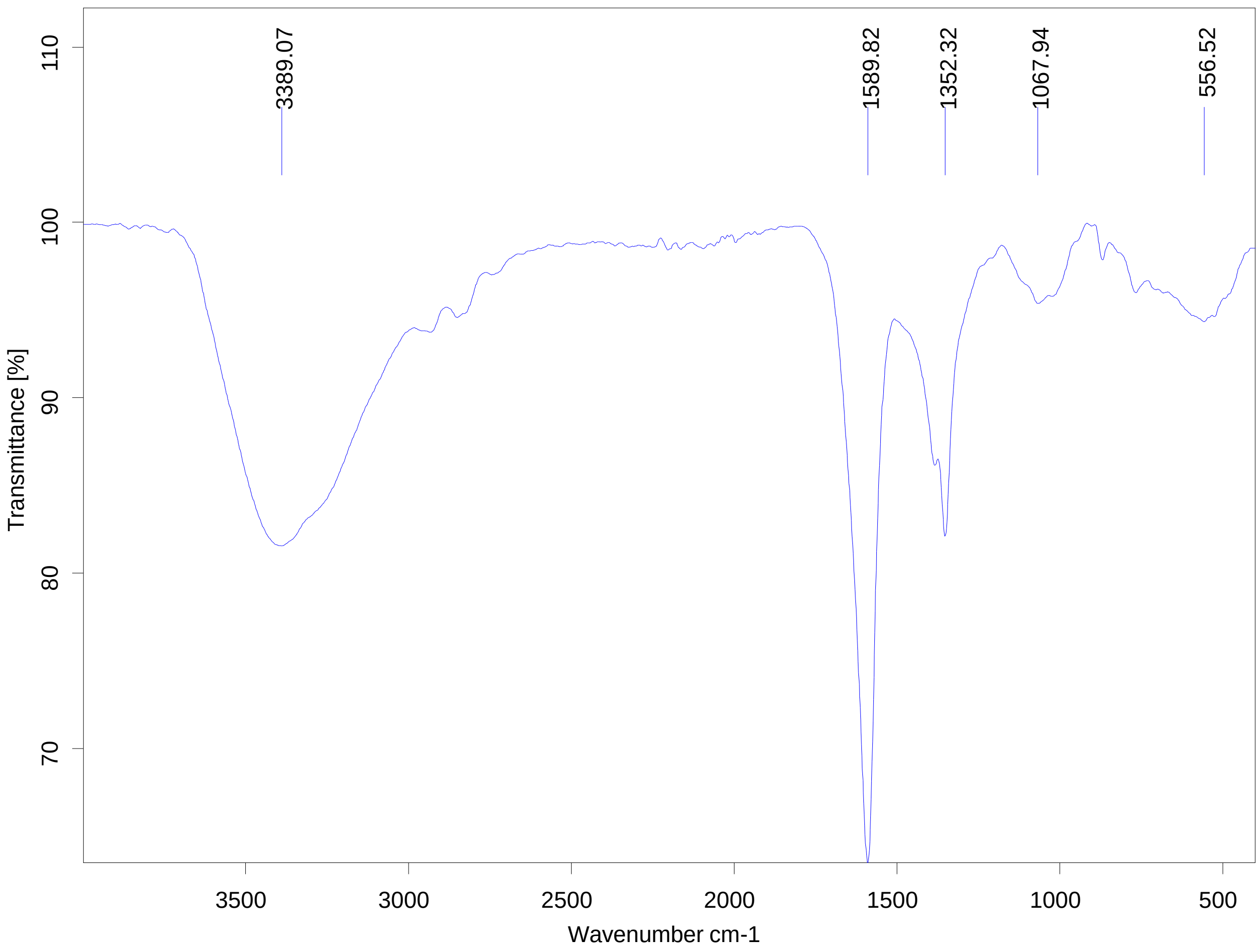

Figure S4. FT-IR spectrum of compound **1**.

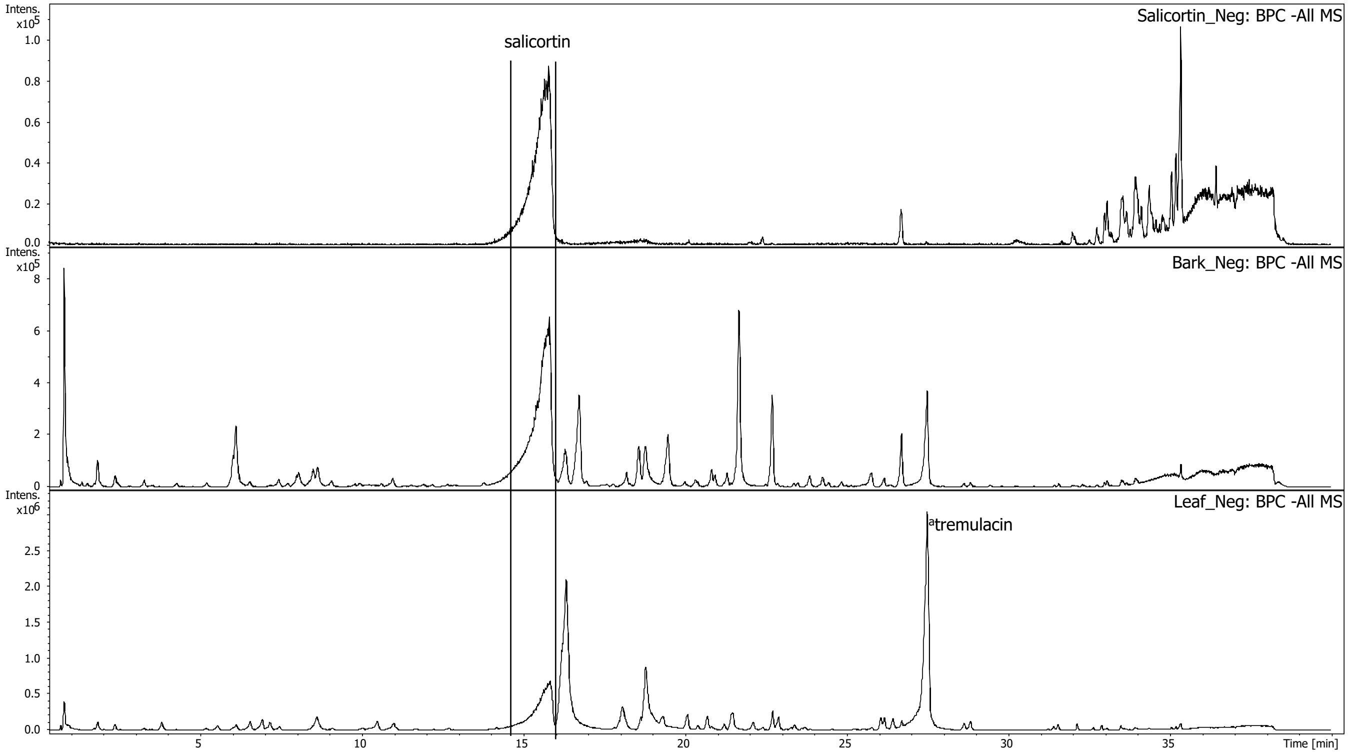

Figure S5. UHPLC-DAD-TIMS-TOF measurement of salicortin (top), MeOH/H_2O (50/50, *v/v*) + 1 % formic acid extract of *Salix purpurea* bark (middle) and MeOH/H_2O (50/50, *v/v*) + 1 % formic acid extract of *Salix purpurea* leaves (bottom) in ESI negative mode.

Table S3. Results of the UHPLC-DAD-TIMS-TOF measurement of salicortin, MeOH/H_2O (50/50, *v/v*) + 1 % formic acid extract of *Salix purpurea* bark and MeOH/H_2O (50/50, *v/v*) + 1 % formic acid extract of *Salix purpurea* leafs in ESI negative mode (cf. Figure S5).

Retention Time [min]	Matrix	Molecular Formula	Theoretical Mass [*m/z*]	Detected Mass [*m/z*]	Mass Error [ppm]	Fragment Ion MS/MS [*m/z*]	CCS [Å²]
15.60	reference	$C_{20}H_{23}O_{10}$	423.1297	423.1298	-0.3	215/155	193.5
15.56	*S. purpurea* bark	$C_{20}H_{23}O_{10}$	423.1297	423.1299	-0.5	215/155	194.2
15.55	*S. purpurea* leaves	$C_{20}H_{23}O_{10}$	423.1297	423.1297	0.0	215/155	193.3
[a]27.45	*S. purpurea* leaves	$C_{27}H_{27}O_{11}$	527.1559	527.1557	0.4	405/155	213.5

Table S4. Full table of NMR data (^{1}H 600 MHz, ^{13}C 151 MHz)[a] of compound **1**.

position[b]	δ_C, type	δ_H (*J* in Hz[c])	HMBC	COSY	ROESY
2	166.8 C				
3	136.8 C				
4	149.7 C				
5	153.5 C				
6	101.1 CH	6.86, d (2.0)	4, 5, 7, 8, 10	8	8, 20, 23, 1'''
7	168.3 C				
8	101.1 CH	7.16, d (2.0)	6, 7, 9, 10	6	6, 22, 2', 6'
9	154.4 C				
10	110.2 C				
11	113.6 C				
12	163.4 C				
13	29.9 CH_2	(*A*) 3.78, ddd (22.7, 3.6, 2.0)	11, 12, 14, 15	(*A*) 13*B*, 14, 15	(*A*) 13*B*, 14, 15, 1''
		(*B*) 4.24, ddd (22.7, 3.6, 2.3)		(*B*) 13*A*, 14, 15	(*B*) 13*A*, 14, 15, 2'', 4''
14	125.0 CH	5.92, dt (10.0, 2.0)	12, 13, 16, 17	13*A/B*, 15	13*A/B*, 15, 20
15	130.1 CH	6.34, dt (10.0, 3.6)	11, 13, 16, 17	13*A/B*, 14	13*A/B*, 14
16	73.6 C				
17	171.7 C				
18	64.9 CH_2	(*A*) 5.35, d (12.2)	17, 19, 20, 24		(*A*) 18*B*, 20, *1*'''
		(*B*) 5.40, d (12.2)			(*B*) 18A, 20, *1*'''
19	126.3 C				
20	131.1 CH	7.22, dd (7.5, 1.7)	18, 22, 23, 24	21	6, 14, 18*A/B*, 21
21	123.7 CH	6.93, td (7.5, 1.0)	19, 20, 22, 23, 24	20, 22, 23	20, 23

22	131.4 CH	7.24, ddd (8.3, 7.5, 1.7)	19, 20, 23, 24	21, 23	8, 21, 23
23	117.4 CH	7.11, dd (8.3, 0.8)	18, 19, 21, 22, 24	22	6, 22, *1*’’’
24	157.4 C				
1’	121.6 C				
2’	118.2 CH	7.72, d (2.3)	2, 1’, 3’, 4’, 6’	6’	8, 6’, 1’’, 2’’
3’	147.3 C				
4’	154.1 C				
5’	117.1 CH	6.98, d (8.6)	2, 1', 3’, 4’	6’	6’, 1’’
6’	126.8 CH	7.91, dd (8.6, 2.3)	2, 2’, 4’	2’, 5’	8, 2’, 5’, 1’’, 2’’
1’’	104.6 CH	4.56, d (7.8)	3, 3’’, 5’’	2’’	13(*A*), 2’, 5’, 6’, 2’’, 3’’, 4’’, 5’’
2’’	75.1 CH	3.60, dd (9.3, 7.8)	1’’, 3’’	1’’, 3’’	13(*B*), 2’, 6’, 1’’, 3’’, 4’’, 5’’
3’’	77.4 CH	3.26, dd (9.0, 9.0)	1’’, 2’’, 4’’	2’’, 4’’	1’’, 2’’, 4’’, 5’’
4’’	71.5 CH	3.17, dd (9.8, 8.9)	3’’, 4’’, 5’’, 6’’	3’’, 5’’	1’’, 2’’, 3’’, 5’’, 6’’(*A*)/(*B*)
5’’	79.1 CH	3.05, ddd (9.8, 6.6, 2.0)	1’’, 3’’, 4’’, 6’’	4’’, 6’’(*A*)/(*B*)	1’’, 2’’, 3’’, 4’’, 6’’(*A*)/(*B*)
6’’	62.5 CH_2	(*A*) 3.38, dd (11.8, 6.6)	4’’, 5’’	(*A*) 5’’, 6’’(*B*)	4’’, 5’’, 6’’(*B*)
		(*B*) 3.68, dd (11.8, 2.0)		(*B*) 5’’, 6’’(*A*)	4’’, 5’’, 6’’(*A*)
1’’’	103.3 CH	4.79, d (7.6)	24, 5’’’	1’’’	6, 18(*A*)/(*B*), 23, 2’’’, 3’’’, 4’’’, 5’’’
2’’’	75.1 CH	3.37, dd (9.2, 7.6)	1’’’, 3’’’	1’’’, 3’’’	1’’’, 4’’’
3’’’	78.2 CH	3.42, dd (9.2, 8.7)	2’’’, 4’’’,	2’’’, 4’’’	1’’’, 4’’’
4’’’	71.6 CH	3.29, dd (9.8, 8.7)	3’’’, 5’’’, 6’’’	3’’’, 5’’’	1’’’, 3’’’, 5’’’, 6’’’(*A*)/(*B*)
5’’’	78.4 CH	3.36-3.39 m	1’’’, 4’’’, 6’’’	4’’’, 6’’’(*A*)/(*B*)	1’’’, 4’’’
6’’’	62.6 CH	(*A*) 3.63, dd (12.0, 5.9)	4’’’, 5’’’	(*A*) 5’’’, 6’’’(*B*)	4’’’, 5’’’, 6’’’(*B*)
	2	(*B*) 3.84, dd (12.0, 2.3)		(*B*) 5’’’, 6’’’(*A*)	4’’’, 5’’’, 6’’’(*A*)

[a] solvent: CD_3OD/TFA-d_1 (95/5, *v/v*); containing TMS (0.01 % *w/v*); δ = 0.0 ppm for ^{1}H and ^{13}C.
[b] for numbering of the carbon atoms, refer to the chemical structure in Figure 3 (assignment of C-H via HSQC data).
[c] for CH_2 groups with diastereotopic protons (*A*) and (*B*) indicate the shielded and deshielded nucleus, respectively.

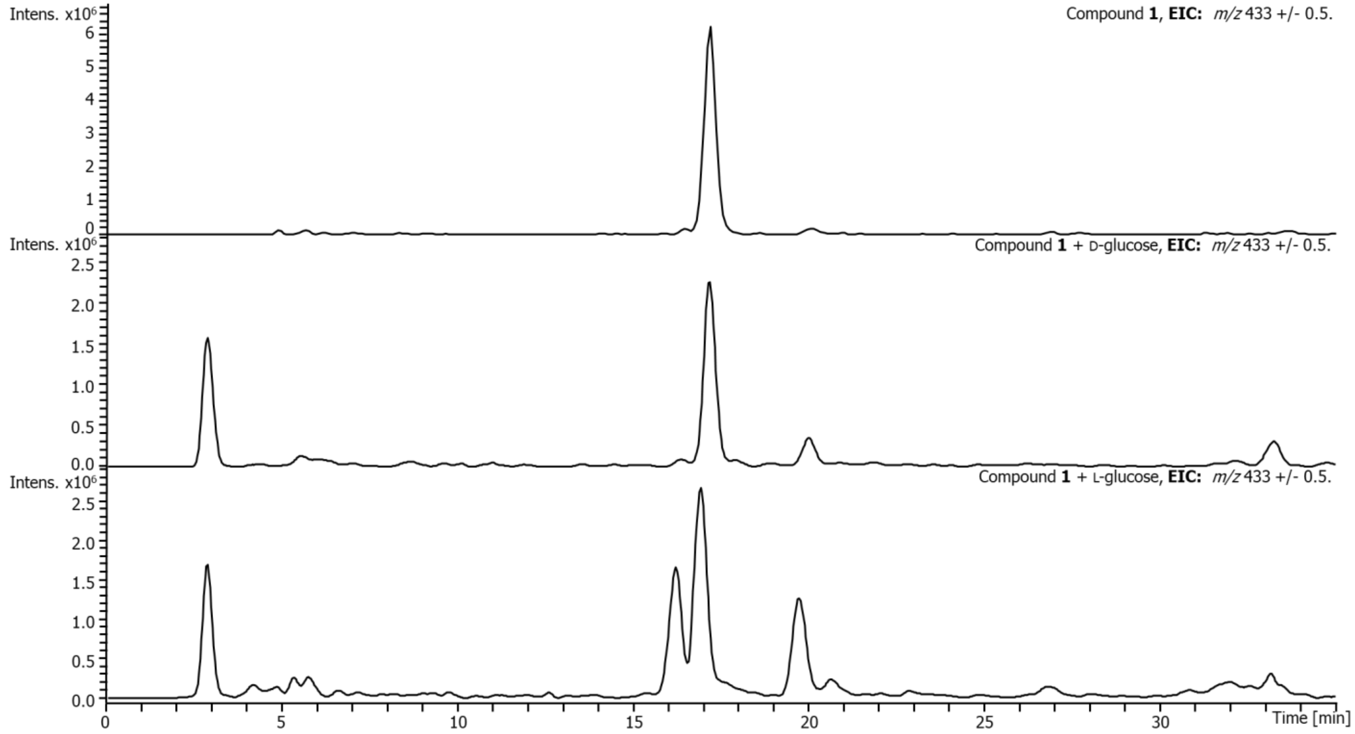

Figure S6. Extracted ion chromatograms (*m/z* 433 +/- 0.5) of derivatized hydrolysate of compound **1** (top), derivatized hydrolysate + derivatized D-glucose (1:1) (middle) and derivatized hydrolysate of compound **1** + derivatized L-glucose (1:1) (bottom) measured in ESI positive mode.

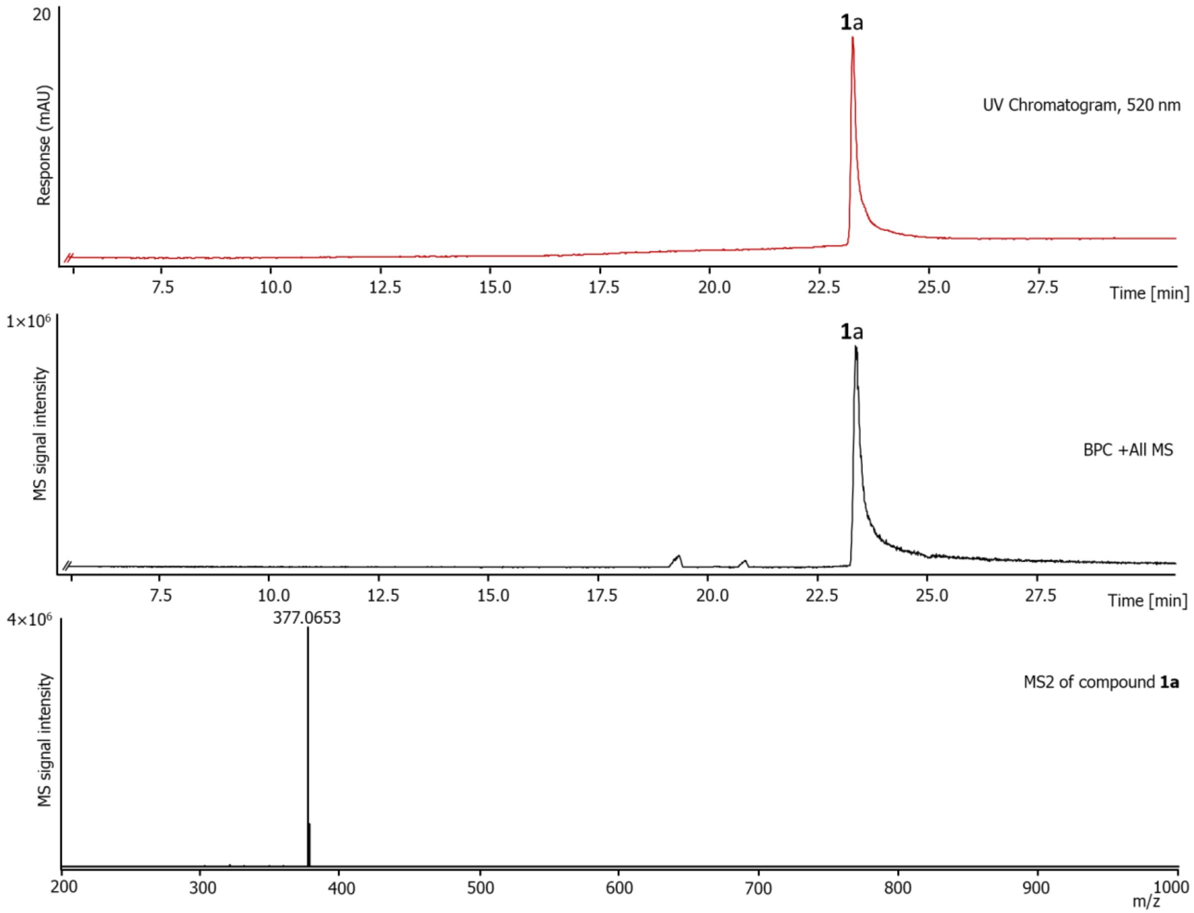

Figure S7. UHPLC-DAD-TIMS-TOF measurement of a the hydrolysate of compound **1** by 2M TFA for 2 h and 90 °C in ESI positive mode and MS2 spectrum of compound **1**a.

Table S5. Results of the UHPLC-DAD-TIMS-TOF measurement of a the hydrolysate of compound **1** by 2M TFA for 2 h and 90 °C in ESI positive mode (cf. Figure S7).

compound Number	Retention Time [min]	Compound Name	UV/Vis max. [nm]	Molecular Formula	Theoretical Mass [*m/z*]	Detected Mass [*m/z*]	Mass Error[ppm]	Fragment Ion MS/MS [*m/z*]	CCS [Å^2]
**1**a	22.79	hydrolysis Artifact **1**a	540	$C_{21}H_{13}O_7^+$	377.0656	377.0653	0.8	-	186.7

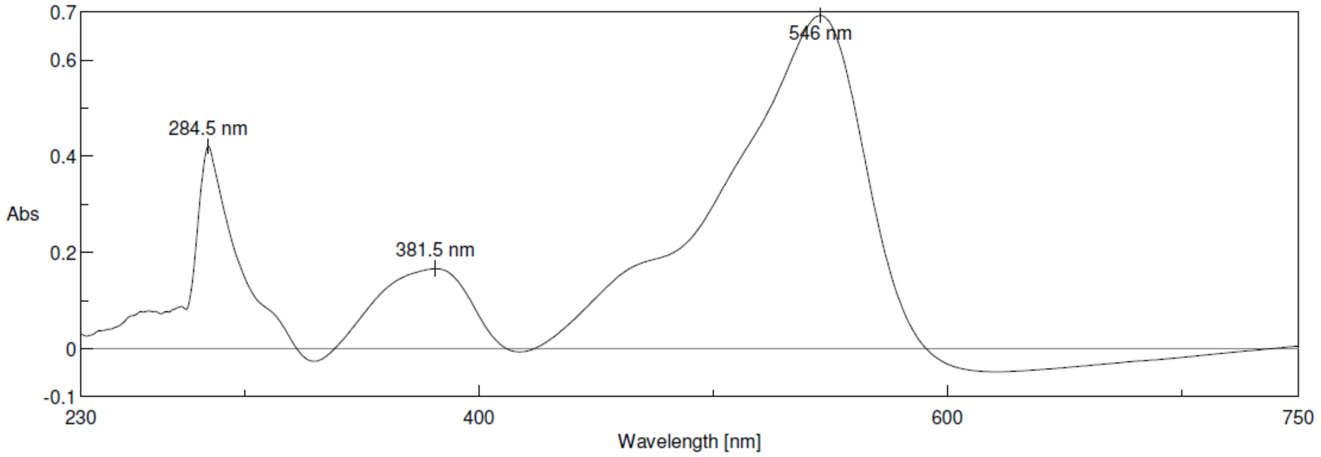

Figure S8. UV/Vis spectrum of compound **1**a in 0.01 % HCl-MeOH.

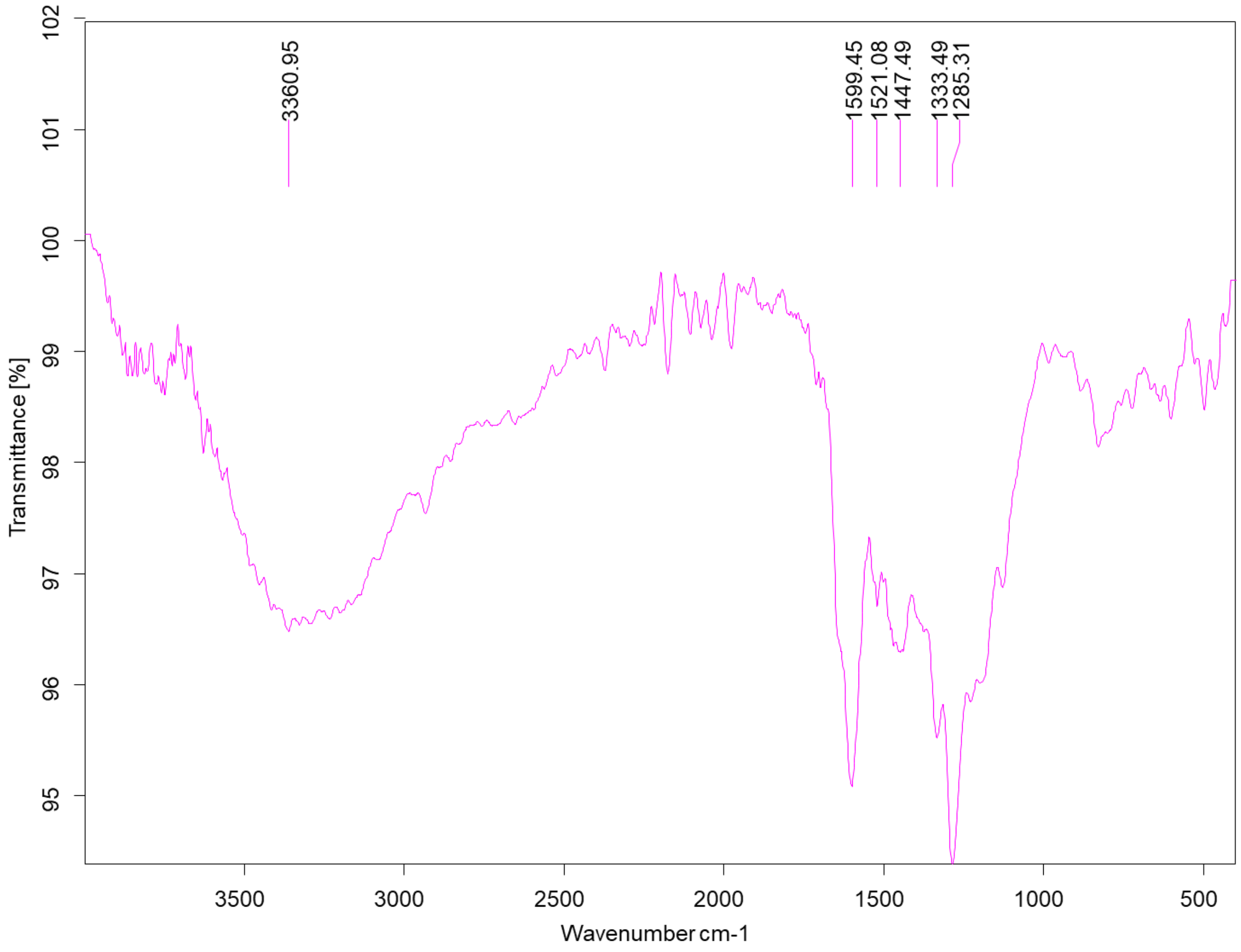

Figure S9. FT-IR spectrum of compound **1**a.

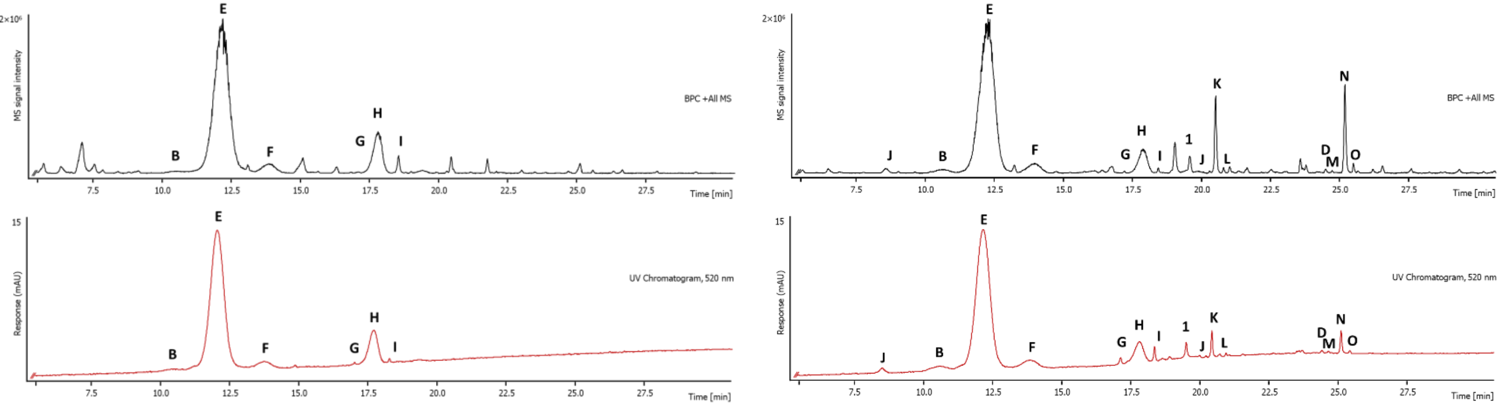

Figure S10. UHPLC-DAD-TIMS-TOF measurement of a strawberry Amberlite™ XAD-7HP extract (left) and combined extract of *Salix purpurea* leaf and strawberry extract after biomimetic formation experiments and purification by Sartobind® IEX MA 75 membrane (right) in ESI positive mode.

Table S6. Results of the UHPLC-DAD-TIMS-TOF measurement of a strawberry Amberlite™ XAD-7HP extract and combined extract of Salix purpurea leaf and strawberry extract after biomimetic formation experiments and purification by Sartobind® IEX MA 75 membrane in ESI positive mode (cf. Figure S10).

**compound Number**	**Retention Time [min]**	**Compound Name**	**UV/Vis max. [nm]**	**Molecular Formula**	**Theoretical Mass[*m/z*]**	**Detected Mass [*m/z*]**	**Mass Error [ppm]**	**Fragment Ion MS/MS [*m/z*]**	**CCS [Å²]**
**J**	8.49	pelargonidin-glucoside-(epi)catechin derivate	n.d.	$C_{36}H_{33}O_{16}$	721.1763	721.1758	0.7	559/313/271	265.7
**B**	10.50	cyanidin-glucoside	n.d.	$C_{21}H_{21}O_{11}$	449.1078	449.1072	1.5	287	202.6
**E**	12.15	pelargonidin-glucoside	276-498	$C_{21}H_{21}O_{10}$	433.1129	433.1224	1.3	271	200.4
**F**	13.87	pelargonidin-rutinoside	276-508	$C_{27}H_{31}O_{14}$	579.1708	579.1704	0.8	433/271	231.0
**G**	17.12	pelargonidin-glucoside-derivate	502	$C_{47}H_{45}O_{23}$	977.2346	977.2344	0.2	545/433/271	287.9
**H**	17.79	pelargonidin-malonyl-glucoside	508	$C_{24}H_{23}O_{13}$	519.1133	519.1129	0.9	433/271	213.6
**I**	18.35	pelargonidin-glucoside-derivate	502	$C_{47}H_{45}O_{23}$	977.2346	977.2340	0.6	545/433/271	285.2
**1**	19.48	compound **1**	506	$C_{41}H_{41}O_{20}$	853.2186	853.2184	0.2	691/423/377	268.0
**J**	20.20	pelargonidin-rutinoside-salicortin	n.d.	$C_{47}H_{51}O_{23}$	983.2816	983.2807	0.9	657/361	291.6
**K**	20.41	pelargonidin-glucoside-salicortin	358-496	$C_{41}H_{41}O_{19}$	837.2237	837.2238	-0.2	675/407/361	262.9
**L**	20.70	pelargonidin-malonyl-glucoside-salicortin	n.d.	$C_{44}H_{43}O_{22}$	923.2240	923.2237	0.4	675/407/361	282.0
**D**	24.39	cyanidin-glucoside-tremulacin	n.d.	$C_{48}H_{45}O_{21}$	957.2448	957.2445	0.3	795/423/377	287.4
**M**	24.63	pelargonidin-rutinoside-tremulacin	n.d.	$C_{54}H_{55}O_{24}$	1087.3078	1087.3075	0.3	779/361	304.7
**N**	25.12	pelargonidin-glucoside-tremulacin	358-496	$C_{48}H_{45}O_{20}$	941.2499	941.2500	-0.1	779/407/361	283.1
**O**	25.40	pelargonidin-malonyl-glucoside-tremulacin	n.d.	$C_{51}H_{47}O_{23}$	1027.2503	1027.2501	0.2	779/361	297.7

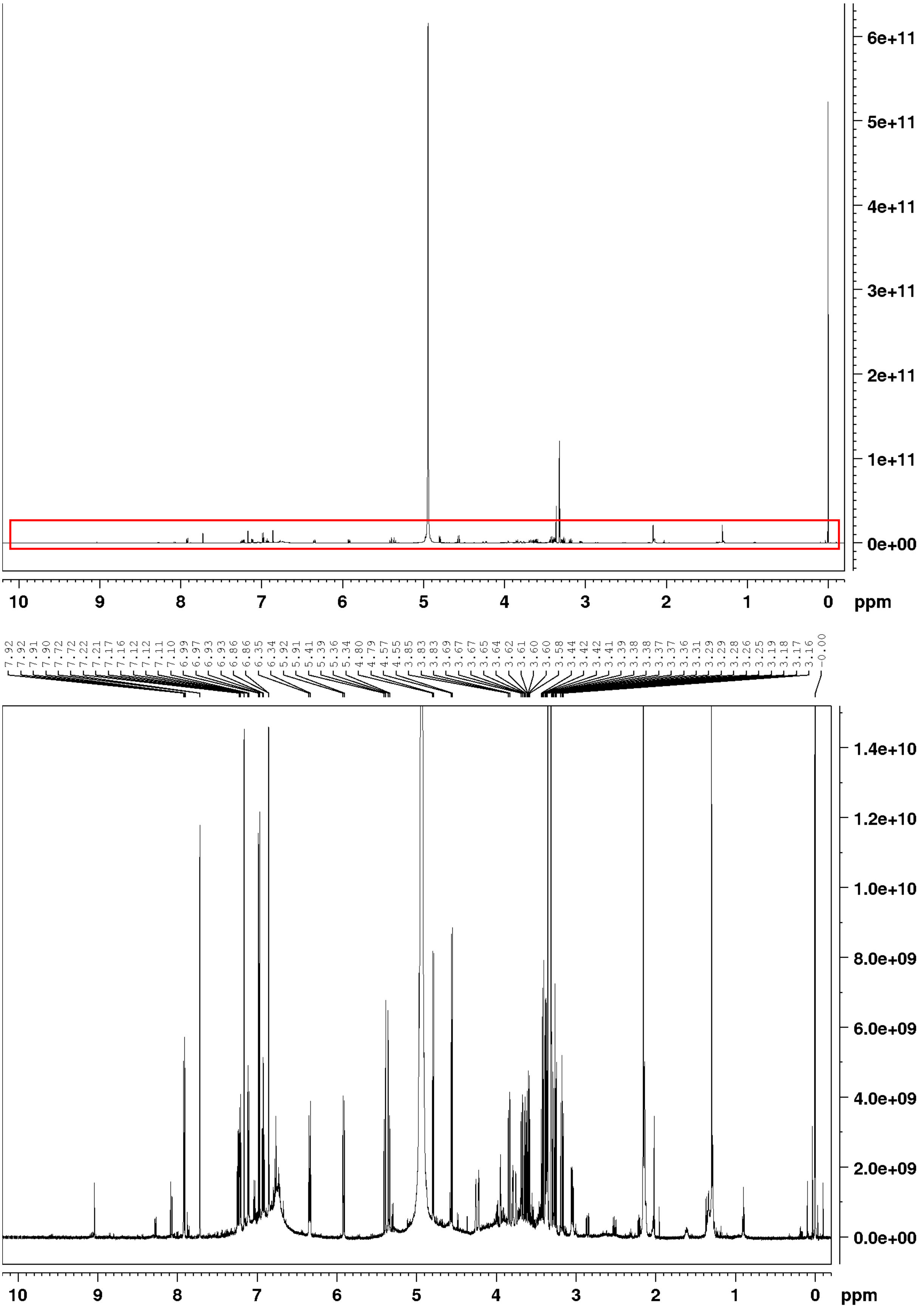

Figure S11. ^{1}H NMR spectrum (CD_3OD/TFA-d, 19:1 *v/v*, 600 MHz) of compound **1**. The top panel shows the full spectrum, while the bottom panel displays a zoomed-in section highlighted by a red box.

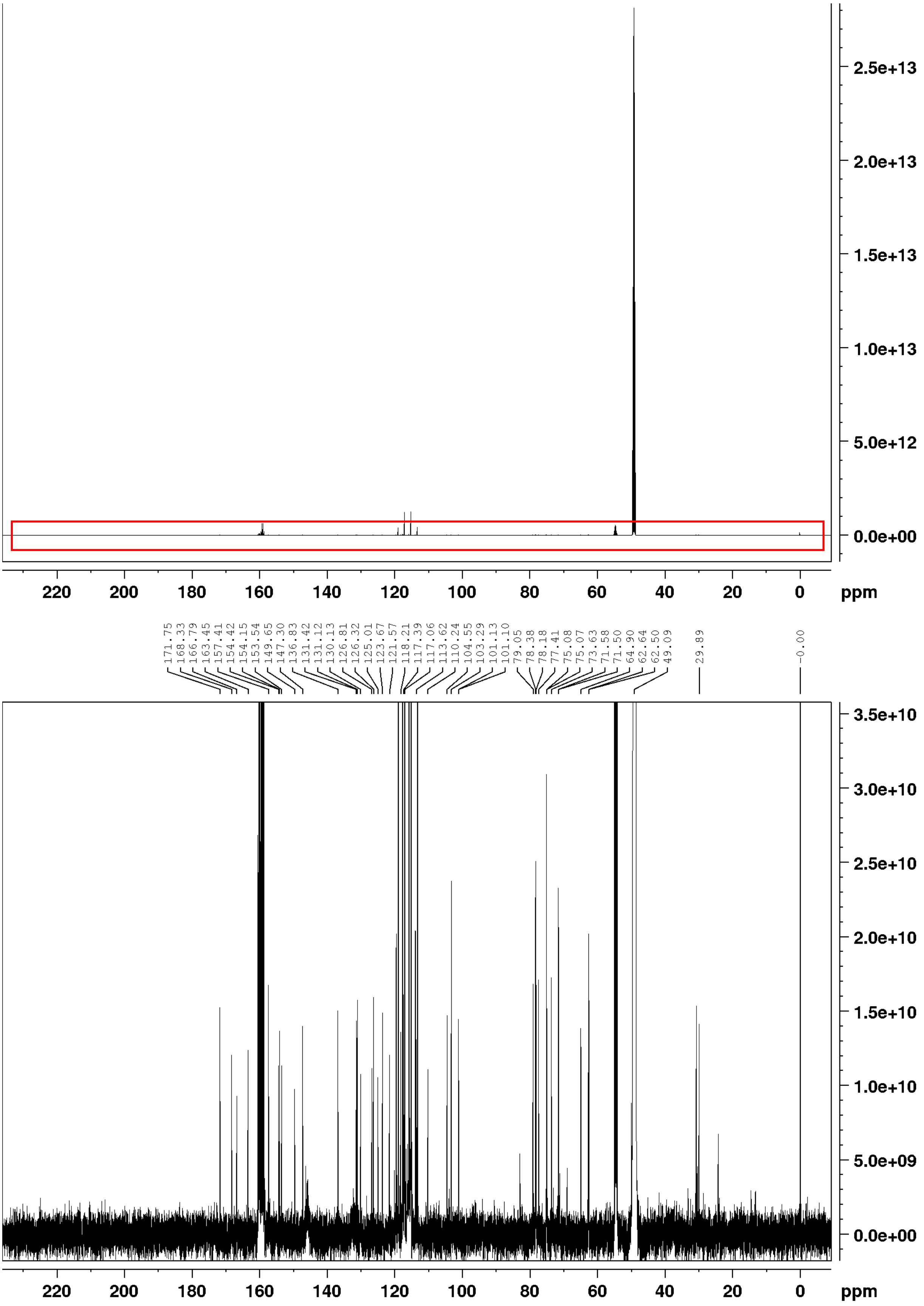

Figure S12. ^{13}C NMR spectrum (CD_3OD/TFA-d, 19:1 *v/v*, 151 MHz) of compound **1**. The top panel shows the full spectrum, while the bottom panel displays a zoomed-in section highlighted by a red box.

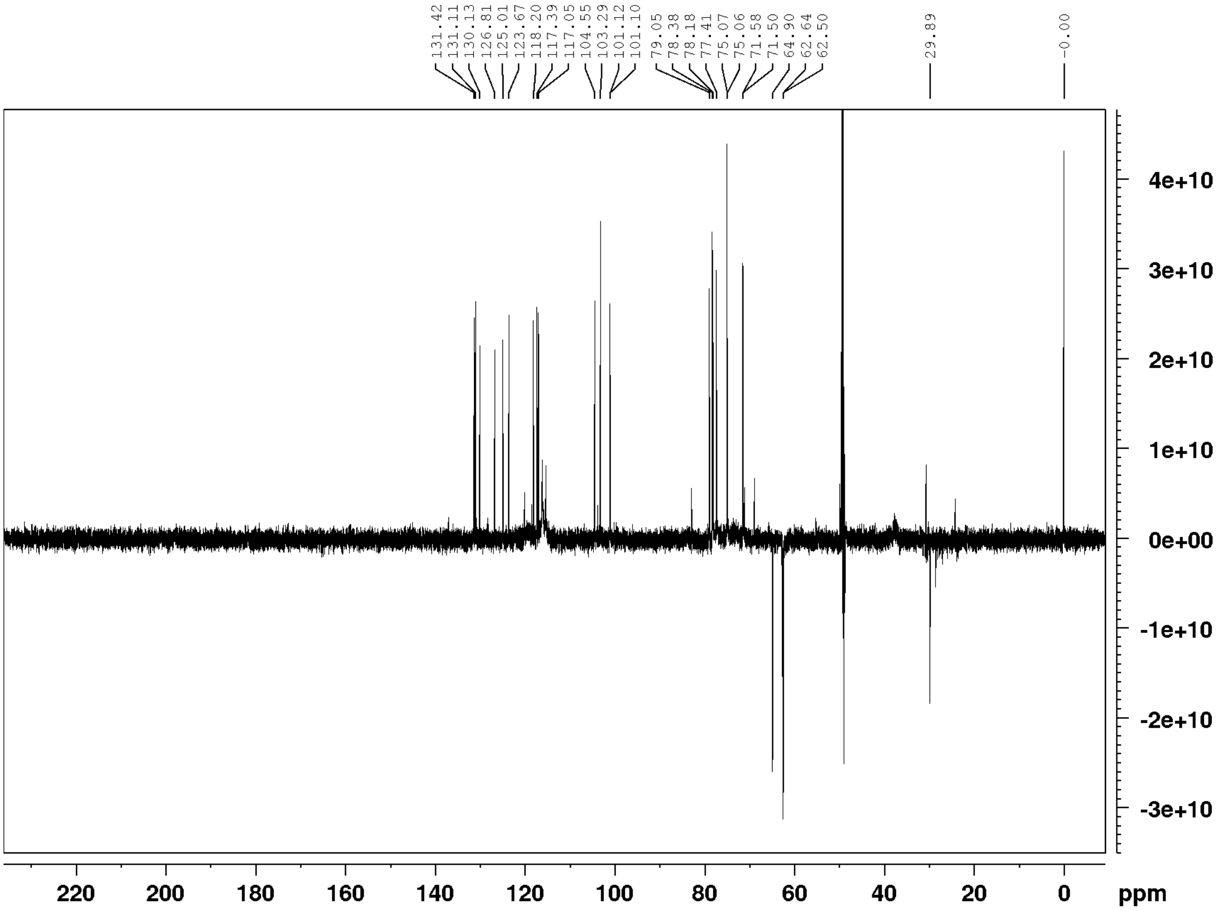

Figure S13. DEPT135 spectrum (CD_3OD/TFA-d, 19:1 *v/v*) of compound **1**.

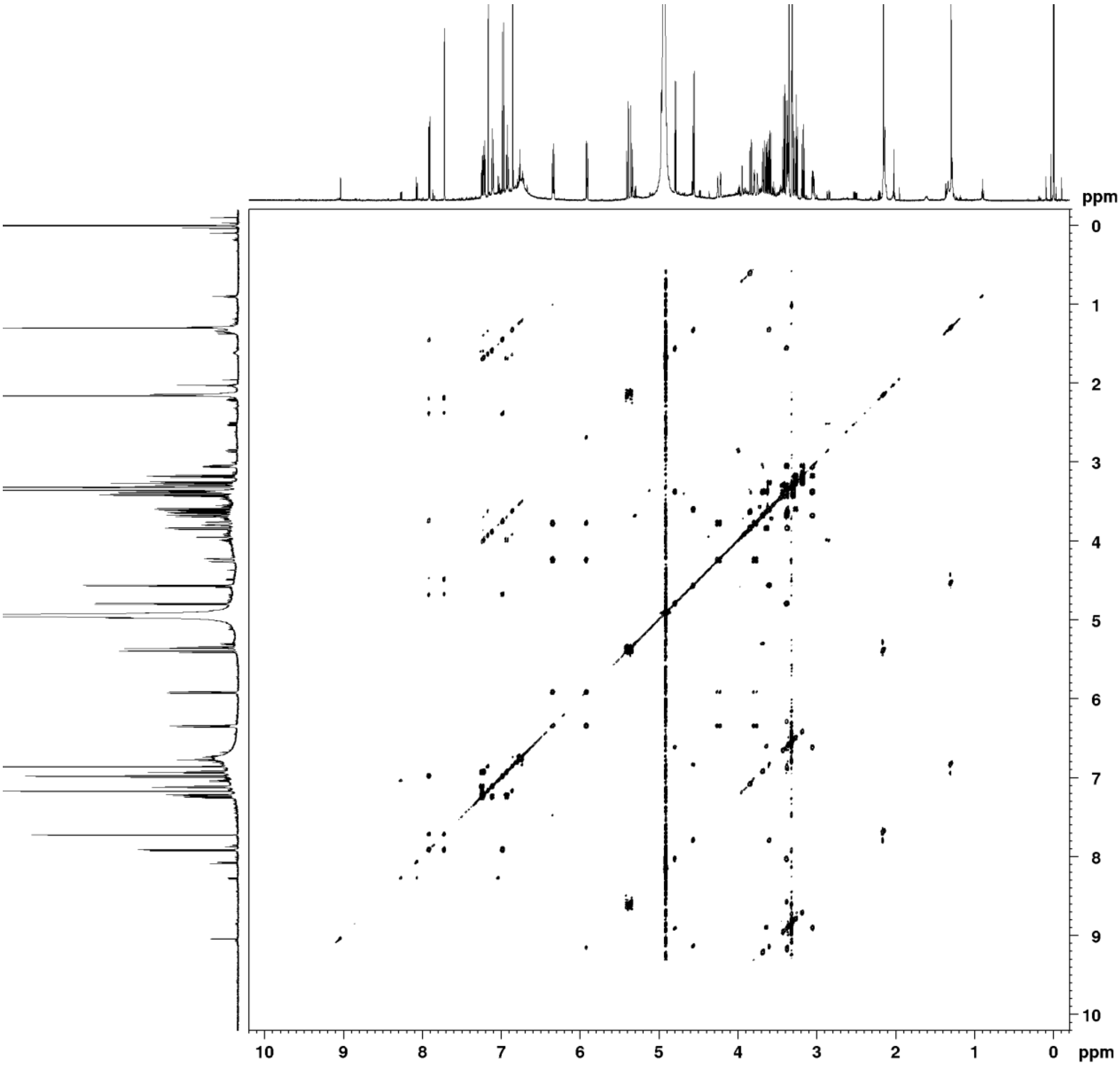

Figure S14. COSY spectrum (CD_3OD/TFA-d, 19:1 *v/v*) of compound **1**.

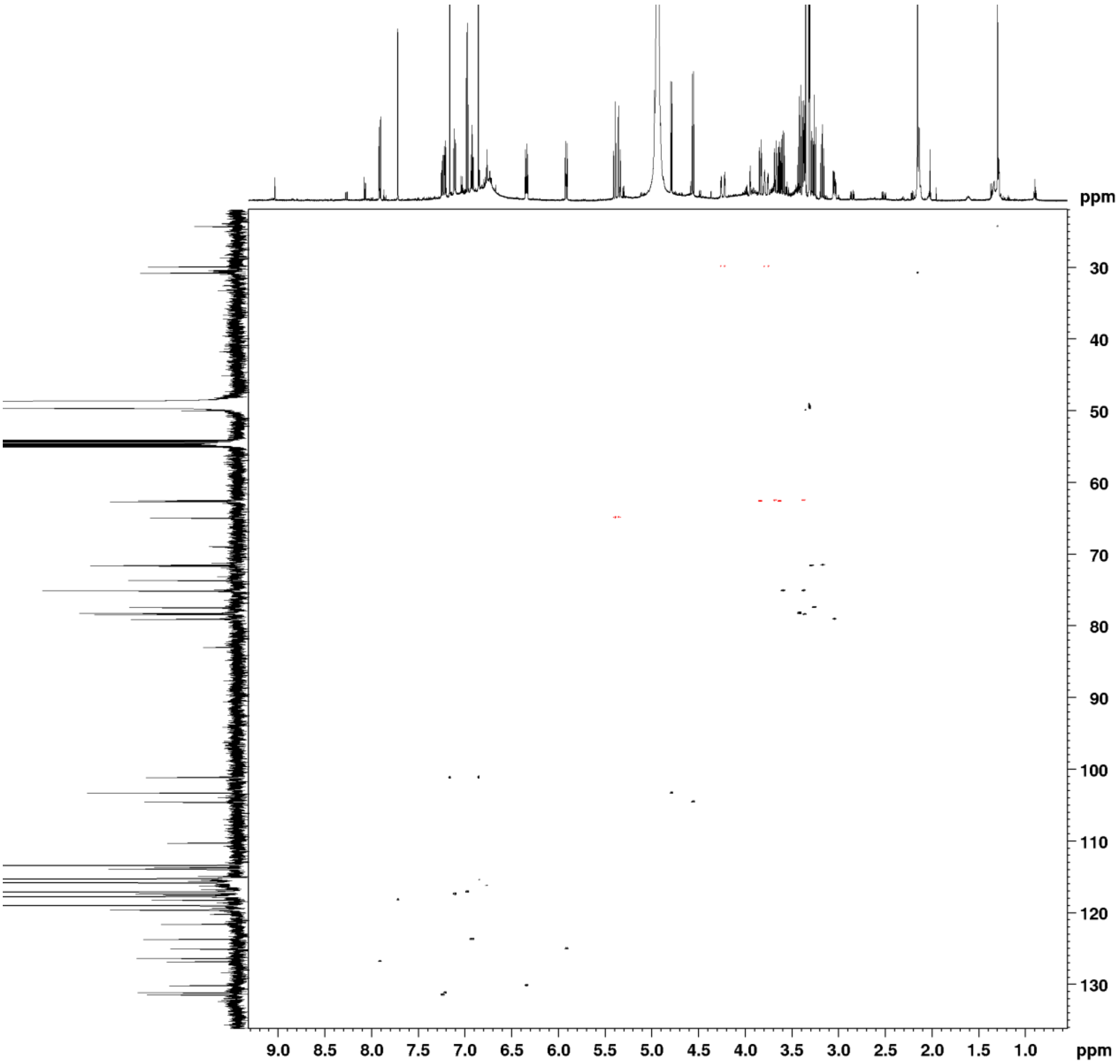

Figure S15. HSQC spectrum (CD_3OD/TFA-d, 19:1 *v/v*) of compound **1**.

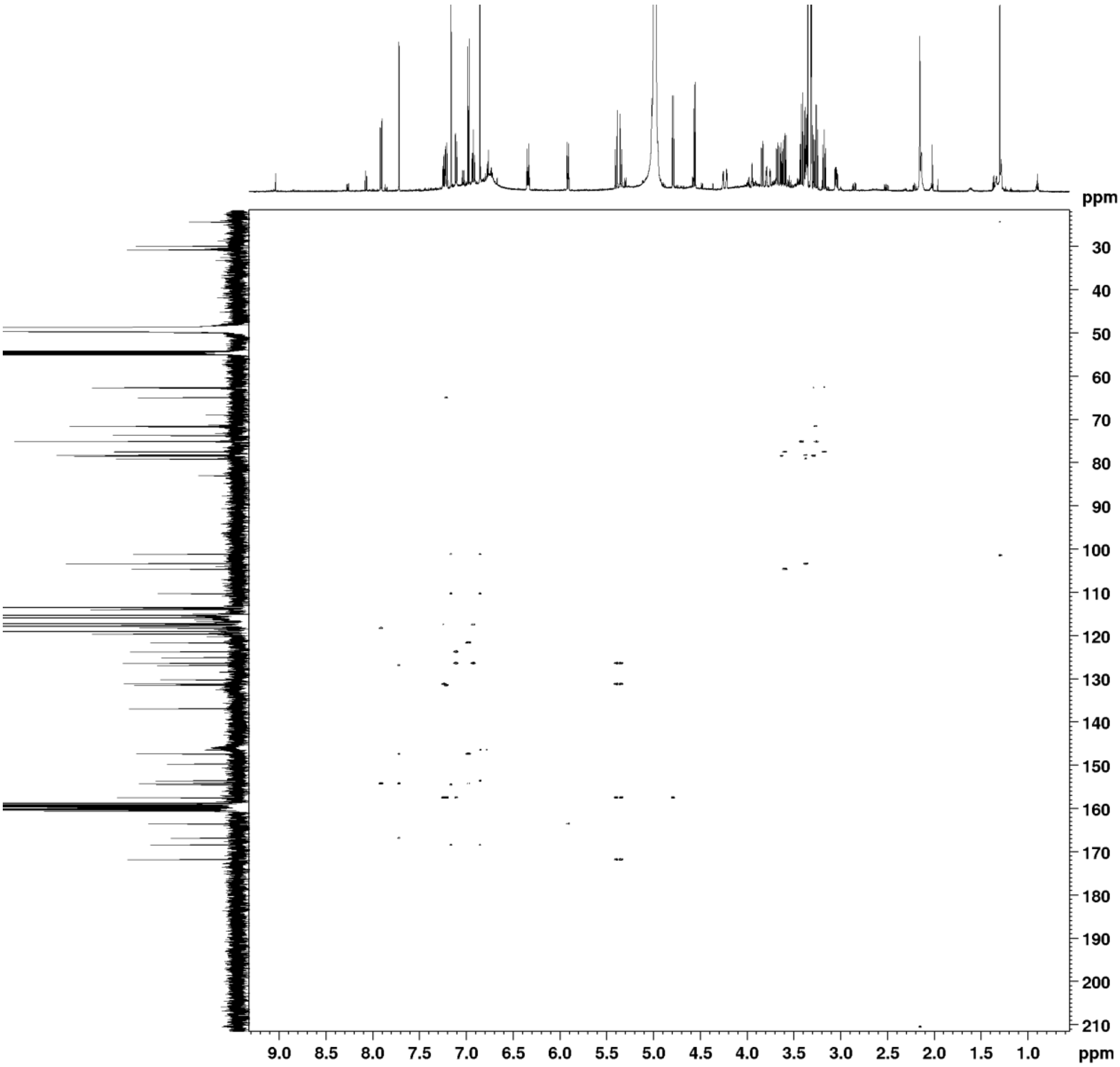

Figure S16. HMBC spectrum (CD_3OD/TFA-d, 19:1 *v/v*) of compound **1**.

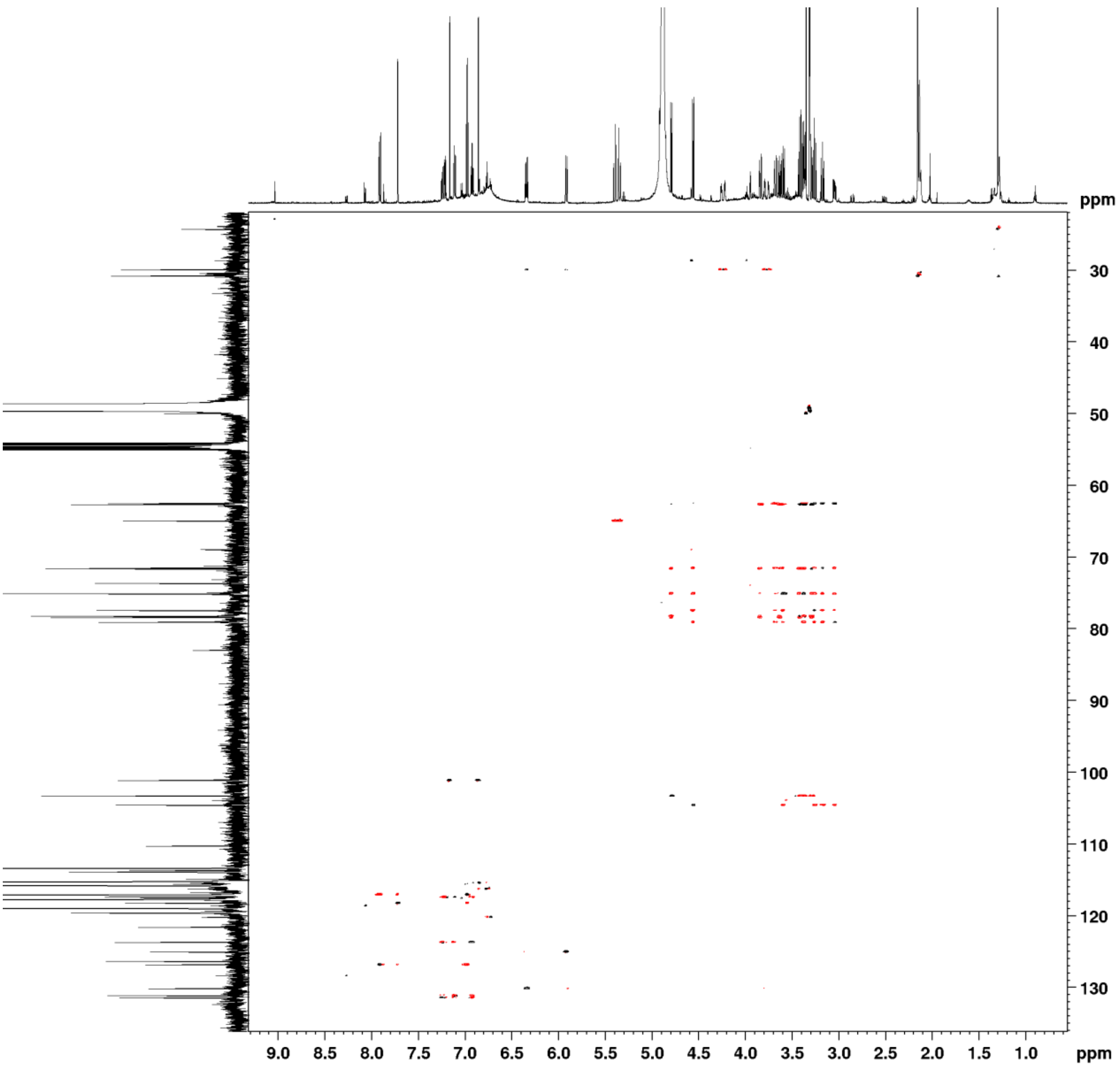

Figure S17. HSQC-TOCSY spectrum (CD_3OD/TFA-d, 19:1 *v/v*) of compound **1**.

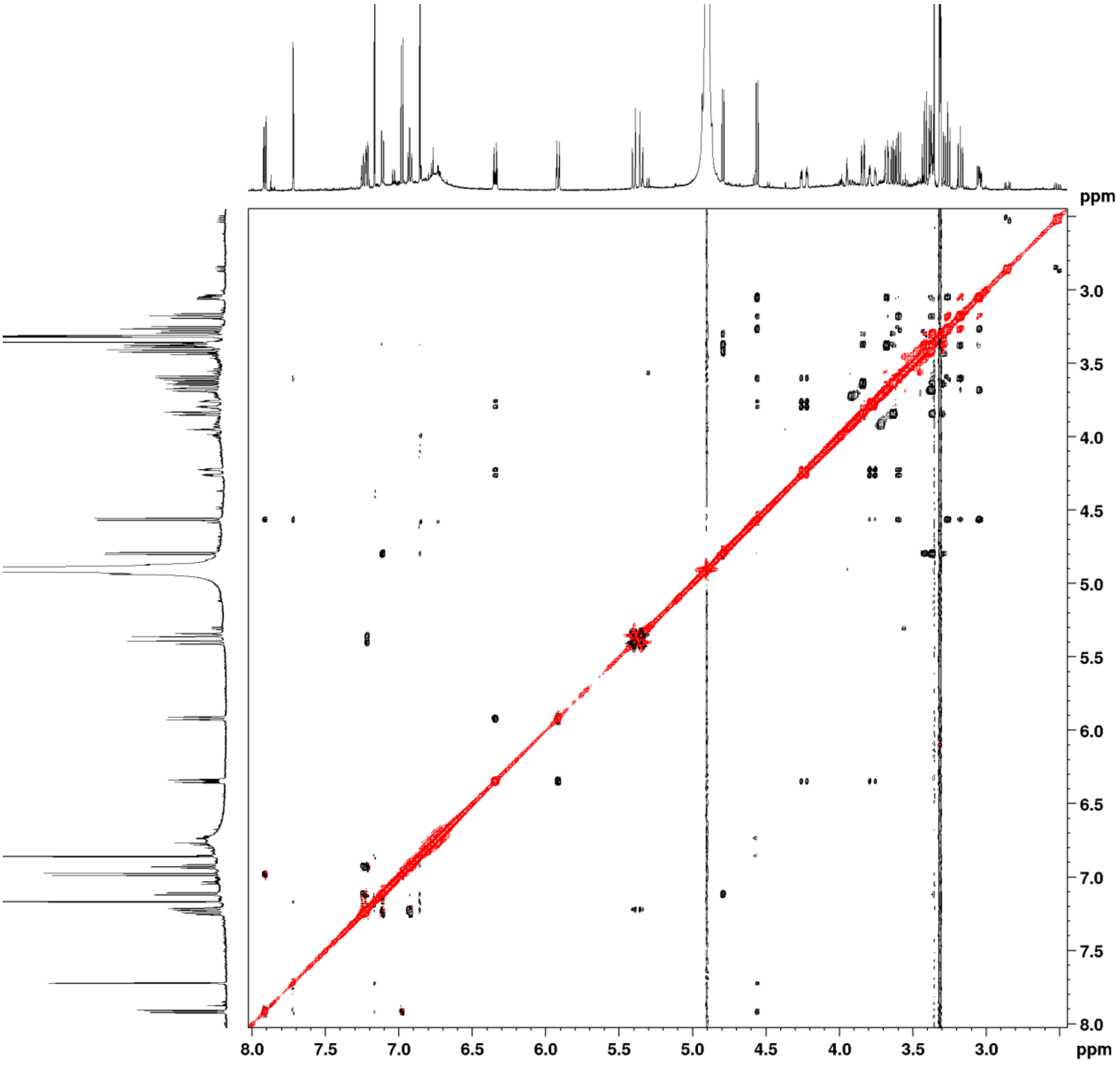

Figure S18. ROESY spectrum (CD_3OD/TFA-d, 19:1 *v/v*) of compound **1**.

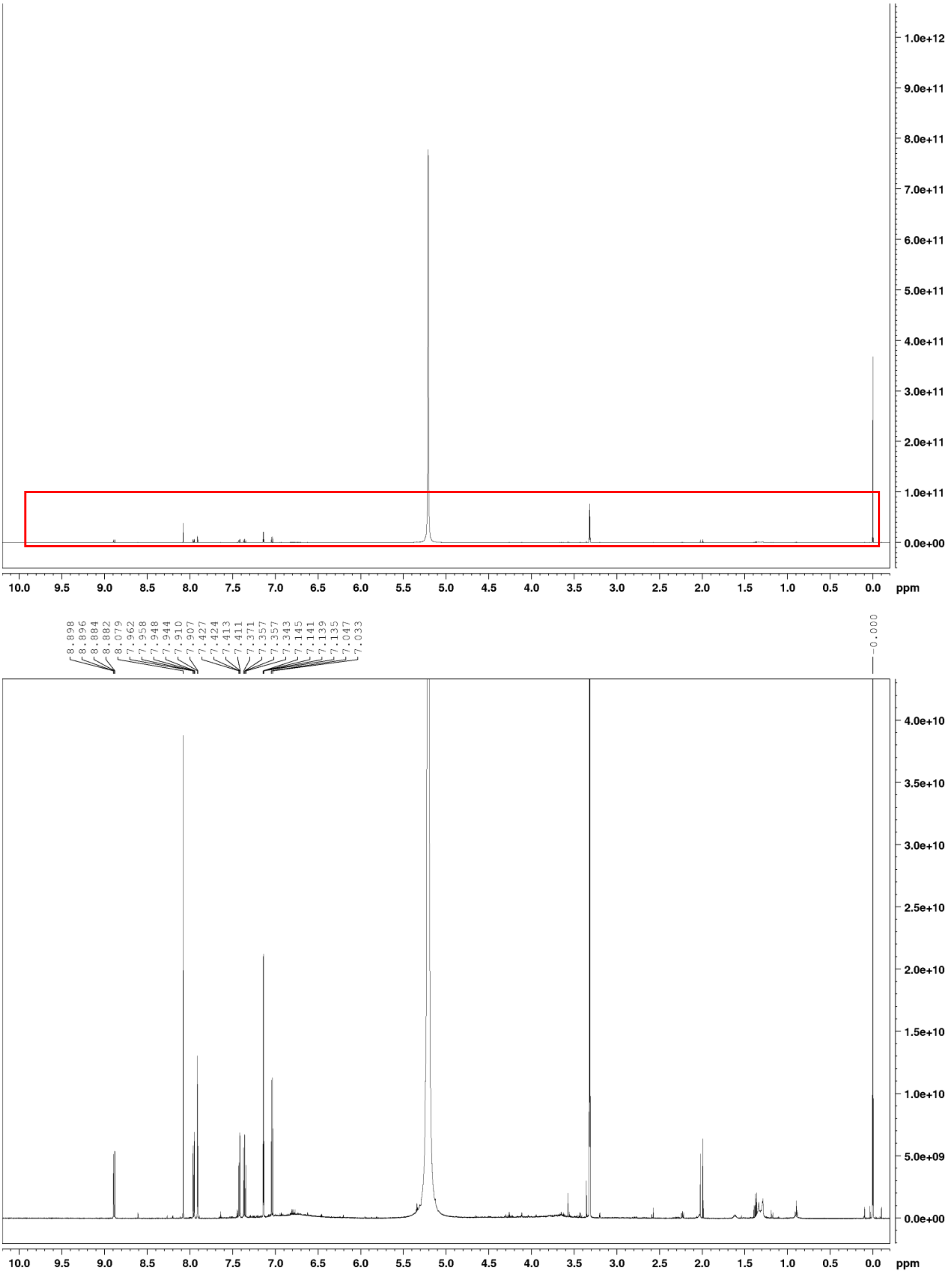

Figure S19. ^{1}H NMR spectrum (CD_3OD/TFA-d, 19:1 *v/v*, 600 MHz) of compound **1**a. The top panel shows the full spectrum, while the bottom panel displays a zoomed-in section highlighted by a red box.

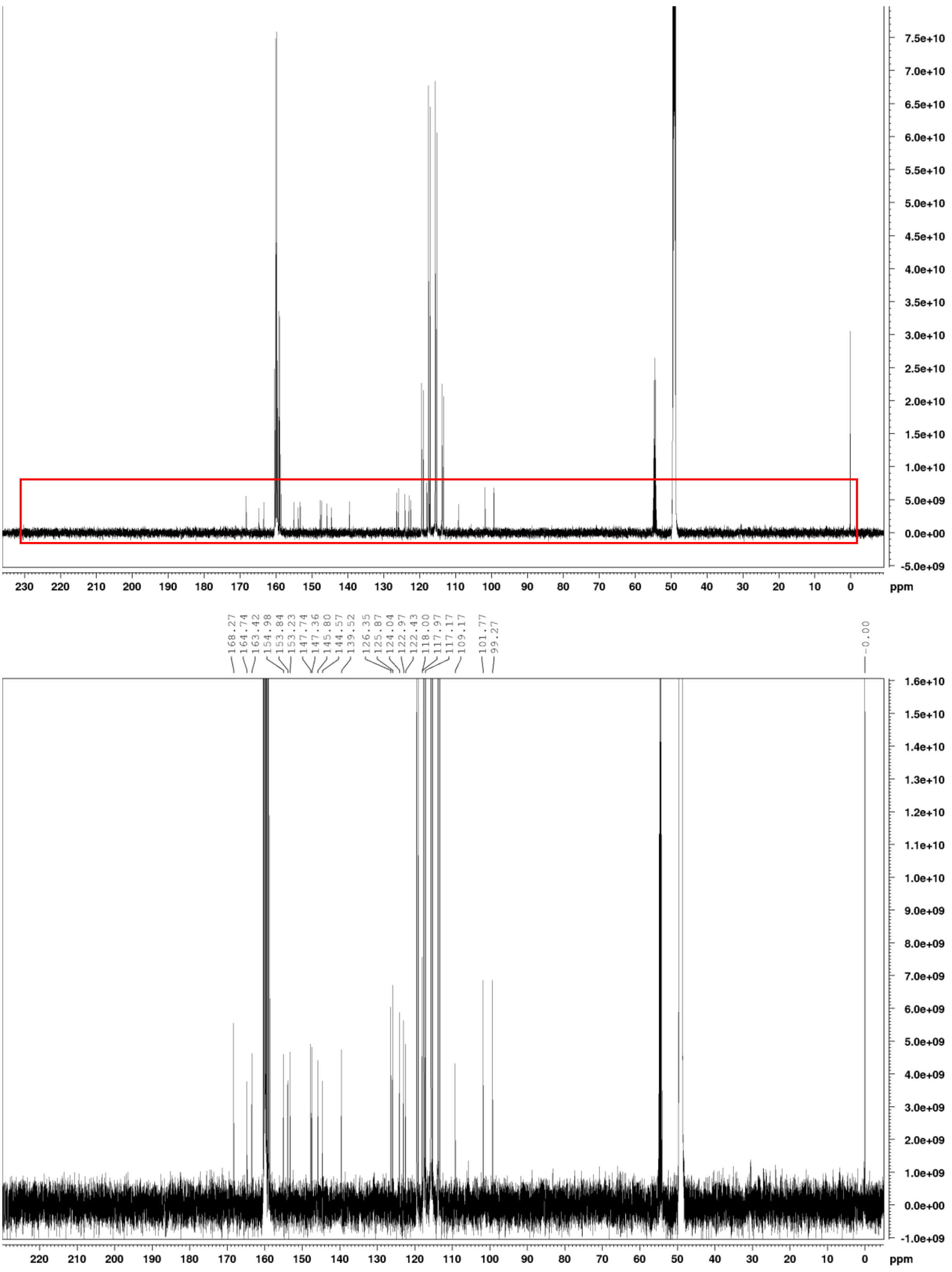

Figure S20. ^{13}C NMR spectrum (CD_3OD/TFA-d, 19:1 *v/v*, 151 MHz) of compound **1**a. The top panel shows the full spectrum, while the bottom panel displays a zoomed-in section highlighted by a red box.

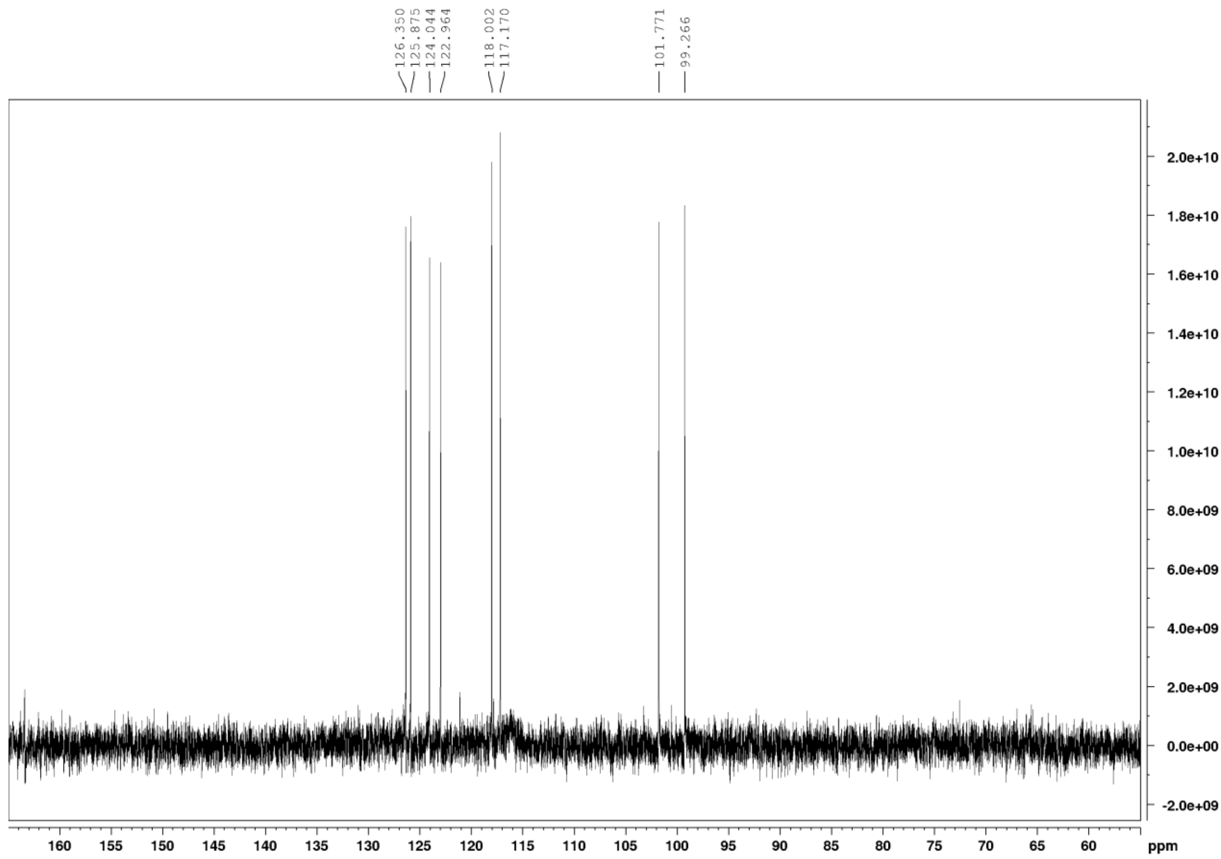

Figure S21. DEPT135 spectrum (CD_3OD/TFA-d, 19:1 *v/v*) of compound **1**a.

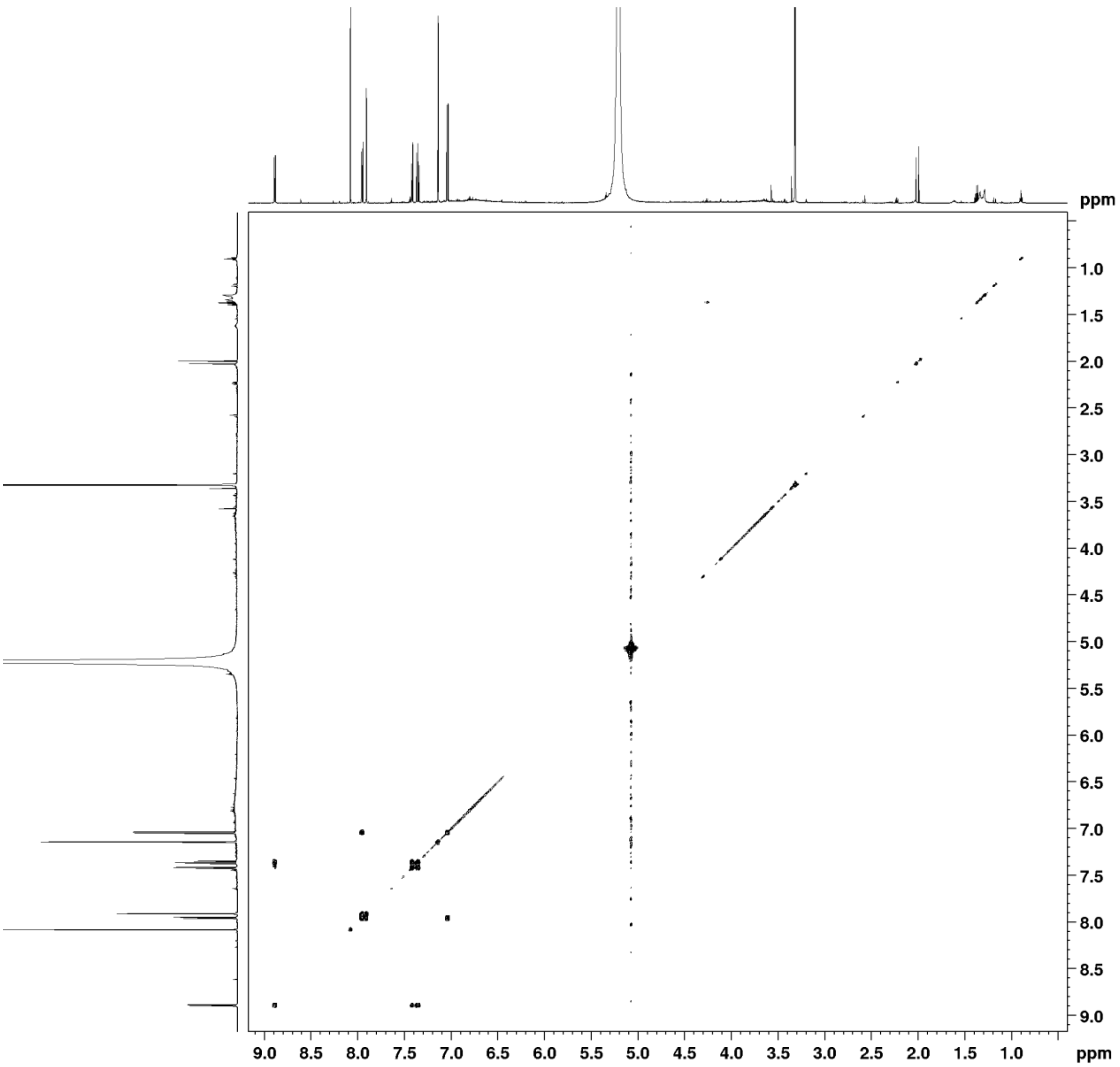

Figure S22. COSY spectrum (CD_3OD/TFA-d, 19:1 *v/v*) of compound **1**a.

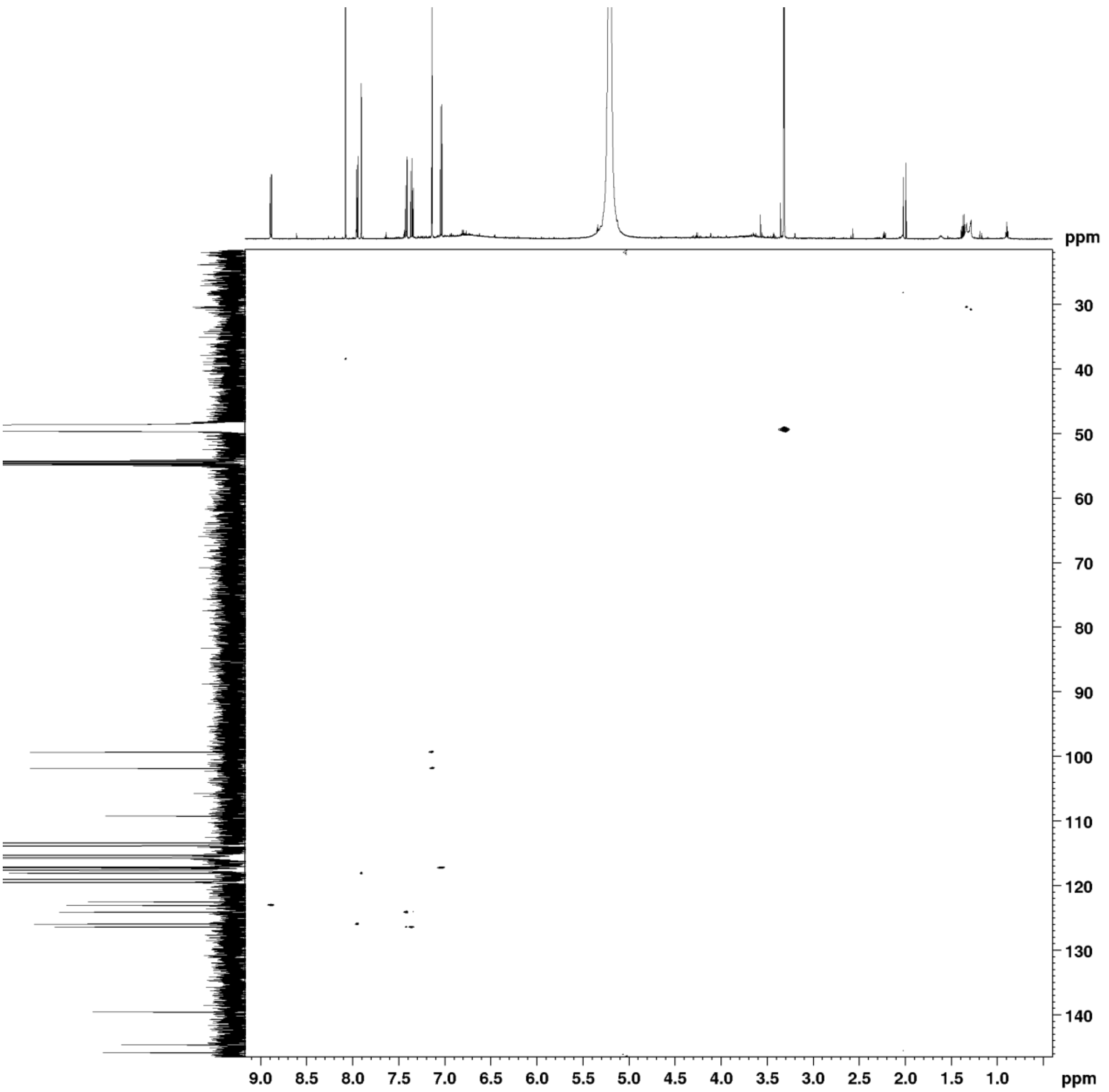

Figure S23. HSQC spectrum (CD_3OD/TFA-d, 19:1 *v/v*) of compound **1**a.

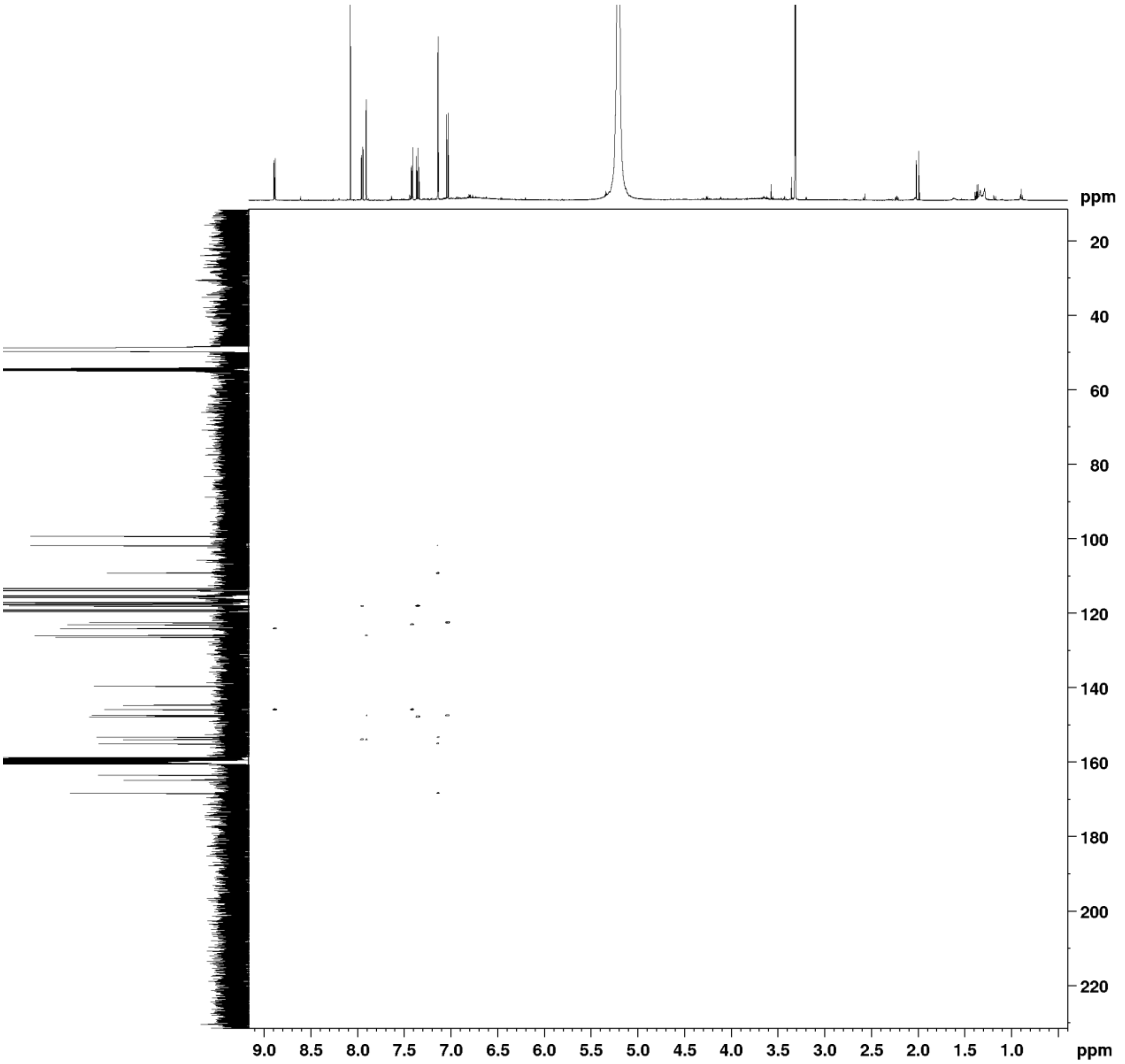

Figure S24. HMBC spectrum (CD_3OD/TFA-d, 19:1 *v/v*) of compound **1**a.
